# Metabolic labeling of the bacterial peptidoglycan by functionalized glucosamine

**DOI:** 10.1016/j.isci.2022.104753

**Published:** 2022-07-12

**Authors:** Yang Xu, Víctor M. Hernández-Rocamora, Joseph H. Lorent, Ruud Cox, Xiaoqi Wang, Xue Bao, Marjon Stel, Gaël Vos, Ramon M. van den Bos, Roland J. Pieters, Joe Gray, Waldemar Vollmer, Eefjan Breukink

**Affiliations:** 1 Membrane Biochemistry and Biophysics, Bijvoet Centre for Biomolecular Research, Department of Chemistry, Utrecht University, Padualaan 8, 3584 Utrecht, the Netherlands; 2 Department of Chemical Biology and Drug Discovery, Utrecht Institute for Pharmaceutical Sciences, Utrecht University, Universiteitsweg 99, 3584 Utrecht, the Netherlands; 3 Centre for Bacterial Cell Biology, Biosciences Institute, Newcastle University, Newcastle Upon Tyne, UK

**Keywords:** Chemistry, Biosynthesis, Biomolecules, Glycobiology

## Abstract

*N*-Acetylglucosamine (GlcNAc) is an essential monosaccharide required in almost all organisms. Fluorescent labeling of the peptidoglycan (PG) on *N*-acetylglucosamine has been poorly explored. Here, we report on the labeling of the PG with a bioorthogonal handle on the GlcNAc. We developed a facile one-step synthesis of uridine diphosphate *N*-azidoacetylglucosamine (UDP-GlcNAz) using the glycosyltransferase OleD, followed by *in vitro* incorporation of GlcNAz into the peptidoglycan precursor Lipid II and fluorescent labeling of the azido group via click chemistry. In a PG synthesis assay, fluorescent GlcNAz-labeled Lipid II was incorporated into peptidoglycan by the DD-transpeptidase activity of bifunctional class A penicillin-binding proteins. We further demonstrate the incorporation of GlcNAz into the PG layer of OleD-expressed bacteria by feeding with 2-chloro-4-nitrophenyl GlcNAz (GlcNAz-CNP). Hence, our labeling method using the heterologous expression of OleD is useful to study PG synthesis and possibly other biological processes involving GlcNAc metabolism *in vivo*.

## Highlights

• Peptidoglycan consists of N-acetylglucosamine, N-acetylmuramic acid, and amino acids
• We developed a one-step synthesis of azide-labeled UDP-N-acetylglucosamine
• In vivo generated azide-labeled UDP-N-acetylglucosamine gets incorporated into peptidoglycan
• Bacteria were fluorescently labeled on N-acetylglucosamine of peptidoglycan

## Graphical abstract

**Figure:**
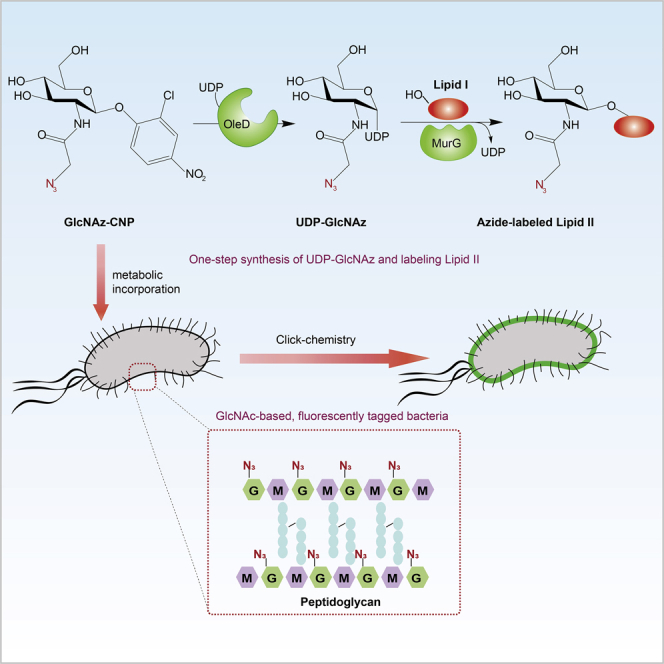

## Introduction

**Figure 1:**
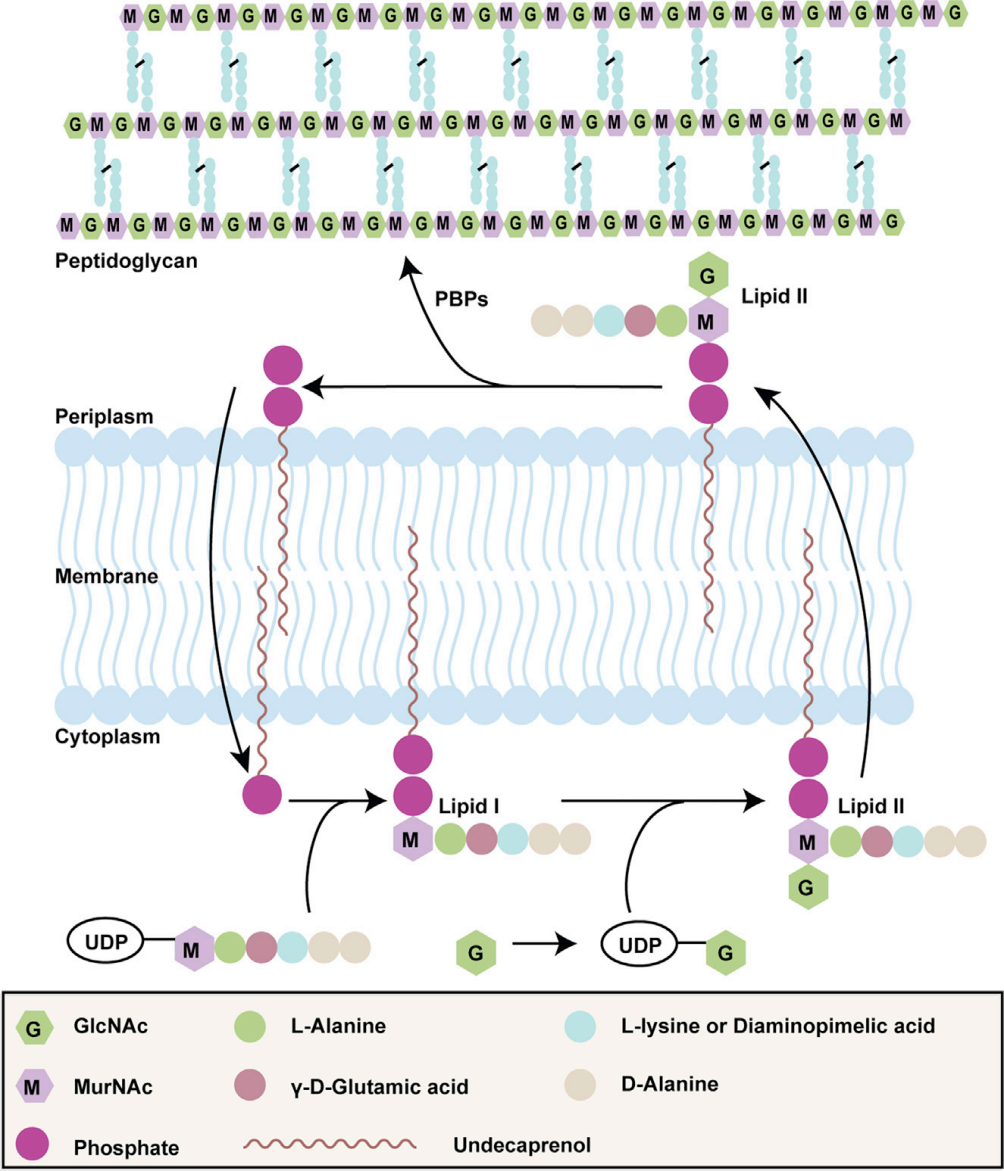
GlcNAc in the peptidoglycan biosynthesis pathway GlcNAc is first converted into UDP-GlcNAc from which it is transferred onto Lipid I to form Lipid II. Lipid II is the precursor in peptidoglycan synthesis utilized by PG synthases to enlarge the PG layer during growth and cell division.

*N*-Acetylglucosamine (GlcNAc or NAG) is an essential monosaccharide required in almost all organisms. In bacteria, GlcNAc is a key component of several important cell envelope molecules, including lipopolysaccharide (LPS) (Raetz and Whitfield, 2002), peptidoglycan (PG) (Vollmer et al., 2008), wall teichoic acid (WTA) (Swoboda et al., 2010), enterobacterial common antigen (ECA) (Kuhn et al., 1988), and poly-*N*-acetylglucosamine (PNGA) (Roux et al., 2015). The PG layer is an essential component of bacteria that maintains the shape of the cell and protects it from bursting due to the high osmotic pressure inside the cytosol (Vollmer et al., 2008; de Pedro and Cava, 2015). The PG layer is composed of a polymer of alternating GlcNAc and *N*-acetylmuramic acid (MurNAc) sugars connected by short peptides attached to the lactoyl acid residue of MurNAc (de Pedro and Cava, 2015; Vollmer et al., 2008). The cytosolic pathway of PG biosynthesis leads to UDP-MurNAc-pentapeptide and UDP-GlcNAc, which on the inner leaflet of the plasma membrane are used for the formation of the final precursor of PG synthesis, undecaprenyl-pyrophosphoryl-MurNAc (pentapeptide)-GlcNAc (Figure 1), called Lipid II. Lipid II is then translocated across the cell membrane where it is used for PG synthesis (Mohammadi et al., 2011; Sham et al., 2014). The final stages of PG synthesis require two reactions: the polymerization of PG glycan chains by glycosyltransferase (GTase) reactions and the formation of the crosslinks between peptides on different chains by transpeptidase (TPase) reactions. Bifunctional class A penicillin-binding proteins (PBPs) can catalyze both GTase and TPase reactions (Figure 1) (Sauvage et al., 2008; Sauvage and Terrak, 2016; Cho et al., 2016; Egan et al., 2017, 2020). The machinery involved in the biogenesis of the PG layer is an ideal target for antibiotics because of its essentiality and the absence of the pathway in mammalian cells. In recent years, several *in vivo* PG-labeling agents have been developed to investigate PG biosynthesis, elucidate the mode of action of antibiotics, and study interactions between pathogenic bacteria and their hosts (Hira et al., 2020). Further work developed procedures for preparing fluorescent D-amino acids, clickable D-amino acids, and D-amino acid dipeptides with bioorthogonal handles providing a means to study PG assembly and turnover in intact bacteria (Kuru et al., 2012, 2015; Bisson-Filho et al., 2017; Monteiro et al., 2015, 2018; Hsu et al., 2017; Hugonnet et al., 2016; Montón Silva et al., 2018) (Figure 1). Fluorescent labeling of PG-precursors and their use *in vitro* and in cells provided important insights into the mode of action of antibiotics and the PG biosynthesis by PBPs and their regulation (Breukink et al., 2003; Hasper et al., 2004, 2006; van Dam et al., 2007, 2009; Olrichs et al., 2011). A recent review on chemical reporters for bacterial glycans provides a nice “panoramic view” of the advances in labeling bacteria (Banahene et al., 2022).

A recent paper reported the synthesis of modified UDP-MurNAc based on a chemoenzymatic synthesis strategy (DeMeester et al., 2018). This strategy takes advantage of the PG-recycling enzymes MurNAc/GlcNAc kinase (AmgK) and MurNAc α-1 phosphate uridylyltransferase (MurU) that can utilize MurNAc mimics containing a bioorthogonal handle (DeMeester et al., 2018; Taylor et al., 2020). The UDP-MurNAc variants were further utilized to fluorescently label PG of the whole cells of bacteria. However, fluorescent labeling of the PG on GlcNAc has been poorly explored. As far as we know, only Sadamoto and coworkers reported specific fluorescent labeling of the PG via a ketone moiety that was introduced into the *N*-acetyl group of GlcNAc (Sadamoto et al., 2008). Yet, the authors did not show the incorporation of ketone-labeled GlcNAc into PG on a molecular level. In our work, we aimed to develop GlcNAc-based probes for studying PG synthesis. This approach would require that the labeled UDP-GlcNAc version is accepted by MurG for the synthesis of Lipid II. The crystal structure of MurG in complex with UDP-GlcNAc reveals that both C3 and C4 hydroxyls of GlcNAc are involved in the recognition of UDP-GlcNAc by MurG (Hu et al., 2003). Thus, these positions are not suitable as bioorthogonal handles. Because the acetyl group does not seem to participate in the binding interface and it has been reported that fluorescent UDP-GlcNAc analogs containing longer linkers at the C2-acetamide were accepted by MurG (Helm et al., 2003; Janetzko and Walker, 2014; Gross et al., 2005), we chose the methyl group of the C2-acetamide as our target site to install an azido group to form UDP-GlcNAz. The azido group would allow the specific labeling with probes via click chemistry. Since PBPs can accept MurNAc analogs of Lipid II as substrates for the synthesis of PG (DeMeester et al., 2018; Liang et al., 2017), we aimed to test if PBPs accept the GlcNAz version of Lipid II.

Traditional chemical synthesis of (unnatural) sugar-nucleotides has been reported over past decades (Ahmadipour and Miller, 2017; Timmons and Jakeman, 2007), whereas enzymatic synthesis has simplified access to these compounds. As previously demonstrated, different methods, some of which use multiple enzyme mixes, have provided access to the synthesis of sugar-nucleotides (Beaton et al., 2009; Guan et al., 2009, 2010; Zhao et al., 2010; Chen et al., 2011; Masuko et al., 2012; Muthana et al., 2015; Li et al., 2016). Moreover, some glycosyltransferases (GTase)-catalyzed reactions are reversible (Zhang et al., 2006), making the formation of nucleotide diphosphate (NDP) sugars thermodynamically unfavorable (Zhang et al., 2006; Quirós et al., 2000; Okada et al., 2009). Hence, it remains challenging to synthesize NDP-sugars from glycoside donors and nucleotide diphosphates. In 2011, Gantt and coworkers reported that aromatic *O*-glycosides can drastically shift the equilibrium of the GTase OleD-catalyzed reactions to overcome this thermodynamic barrier (Gantt et al., 2011). A variant of OleD (OleD TDP-16) was shown to catalyze the formation of NDP-sugars from a 2-chloro-4-nitrophenyl glycoside donor (Gantt et al., 2013). Moreover, this OleD variant was capable of catalyzing the formation of natural and non-natural UDP-activated sugars (Gantt et al., 2013; Zhang et al., 2014). Herein, we explore this approach for the synthesis of UDP-GlcNAz in one step by the enzyme OleD TDP-16. We demonstrate the synthesis of UDP-GlcNAz on a preparative scale and show that it is accepted as a substrate by Lipid II-synthesizing enzymes, enabling the efficient synthesis of GlcNAz-containing Lipid II, which was subsequently fluorescently labeled. We further show that this Lipid II variant was not accepted as a GTase substrate by PBPs which nevertheless incorporated fluorescently labeled GlcNAz-Lipid II into PG by TPase reactions. Finally, we succeeded in labeling the PG in *Escherichia coli* cells expressing OleD by feeding them with 2-chloro-4-nitrophenyl-*N*-azidoacetylglucosamine (GlcNAz-CNP) and subsequent analysis indicated that the labels were incorporated into the peptidoglycan layer.

## Results and discussion

### Chemical synthesis of azide-labeled D-glucosamine

**Figure 2:**
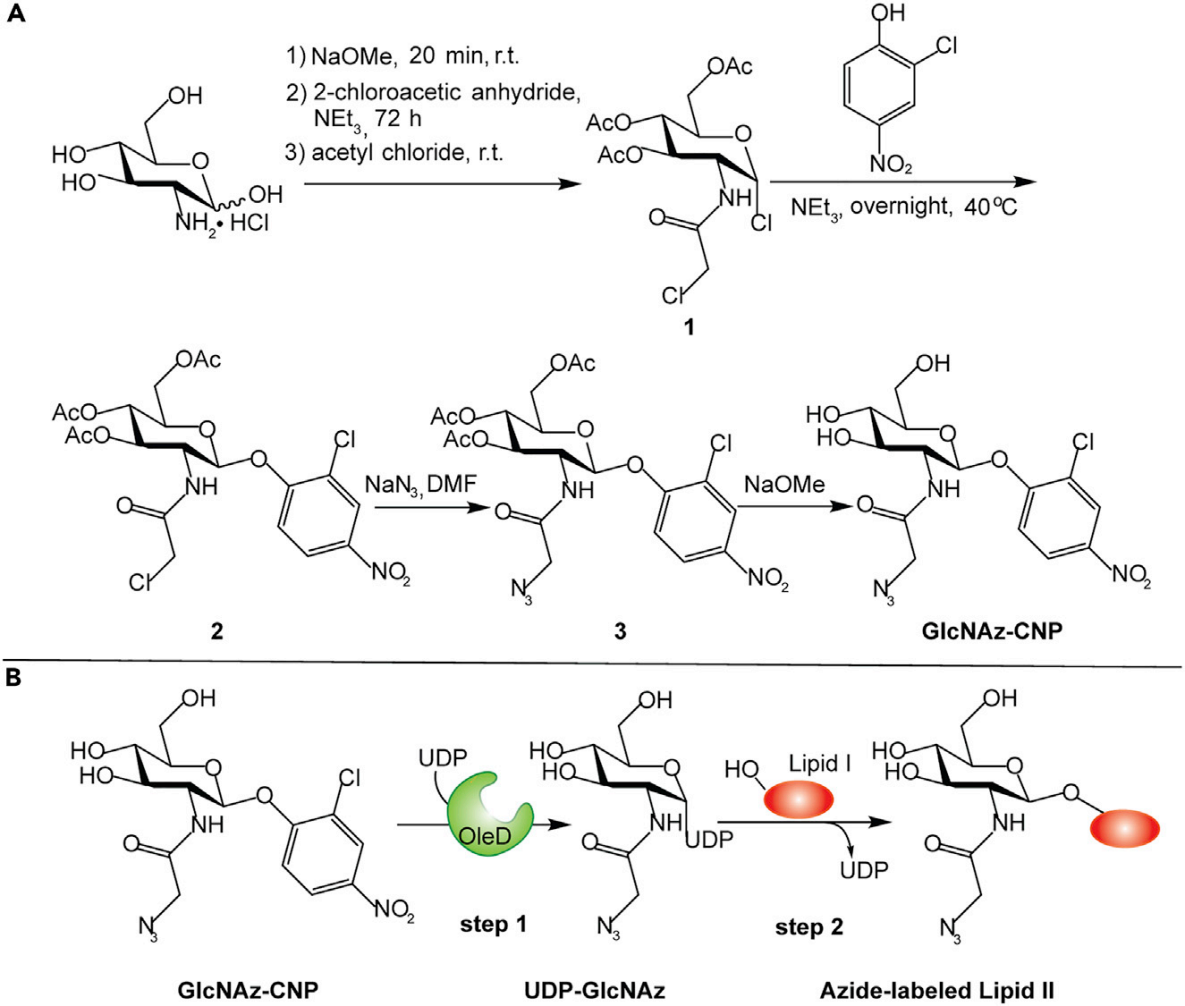
Chemical synthesis scheme of GlcNAz-CNP and its use in the synthesis of UDP-GlcNAz and labeled Lipid II (A) Chemical synthesis scheme of GlcNAz-CNP. Reagents and conditions: a) i NaOMe, 20 min, r.t. ii 2-chloroacetic anhydride, NEt_3_, 72 h iii AcCl, r.t (64%); b) NEt_3_, 40°C, 14 h (71%); c) NaN_3_, DMF (90%), d) NaOMe, methanol (83%). (B) One-step synthesis of UDP-GlcNAz by the glycosyltransferase (OleD) and its use in the synthesis of labeled Lipid II. The glycosyltransferase-catalyzed reaction between GlcNAz-CNP and UDP by OleD generated UDP-GlcNAz (step 1). UDP-GlcNAz was further utilized by MurG to attach GlcNAz to Lipid I (red oval), producing azide-labeled Lipid II (step 2).

We first set out to optimize the synthesis of the activated sugar (GlcNAz-CNP) such that it would allow the facile synthesis of relatively large amounts. To synthesize an aromatic glycoside, the targeted hydroxyl group of the sugar needs to be activated by introducing a halide (Demchenko, 2008). In this way, the halide can be substituted with acceptors such as phenols to generate the desired glycoside. In a previous study, 2-chloro-4-nitrophenyl-β-D-glucopyranoside was synthesized using the reaction between a glycosyl bromide and 2-chloro-4-nitrophenol, where the glycosyl bromide was synthesized using a two-step procedure (Gantt et al., 2011). Here, we first introduce a chloroacetyl group on the amine of glucosamine to enable the installation of the azido group in the third step (Figure 2A). In short, glucosamine hydrochloride was first neutralized by sodium methoxide (NaOMe) to give glucosamine. Then, the free amine of glucosamine was allowed to react with 2-chloroacetic anhydride to install a chloroacetyl group, that subsequently reacted with acetyl chloride, resulting in the protection of the hydroxyl groups at C3, C4, C6, and substitution at C1 to give compound **1**. By using acetyl chloride as solvent and reaction substrate to produce compound **1**, the acetylation and chloride-substitution are achieved in one step. The resulting product reacted with 2-chloro-4-nitrophenol in the presence of triethylamine to the 2-chloro-4-nitrophenyl glycoside. The final product, 2-chloro-4-nitrophenyl GlcNAz (GlcNAz-CNP), was synthesized by introduction of the azido group and removal of the acetyl groups.

### Chemoenzymatic synthesis of UDP-GlcNAz

**Figure 3:**
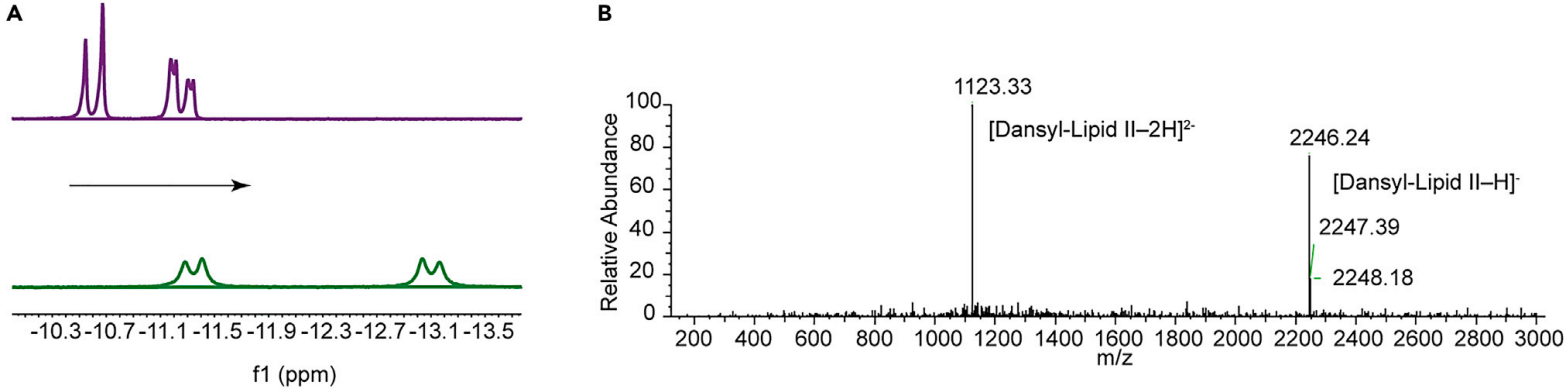
UDP-GlcNAz synthesis and its incorporation into Lipid II (A) ^31^P NMR spectra of UDP before (top, in purple) and after reaction to form UDP-GlcNAz (bottom, in green). A chemical shift of 1–2 ppm toward high field (arrow) compared to UDP indicates conversion to UDP-GlcNAz. (B) Mass spectrum in negative ion mode of dansyl-GlcNAz-Lipid II. The *m*-Dap version of dansyl-GlcNAz-Lipid II was generated by click chemistry (see STAR Methods). The mass of dansyl-GlcNAz-Lipid II as determined in the negative mode (2247.24 Da) is in accordance with the calculated exact mass of 2246.15 Da.

Next, we used the purified OleD variant TDP-16 for the synthesis of UDP-GlcNAz (Figure 2B). For this, the enzyme was incubated with a mixture containing UDP, 2-chloro-4-nitrophenyl GlcNAz (GlcNAz-CNP), and MgSO_4_. The glycosyltransferase reaction was followed by measuring the appearance of yellow color due to the production of 2-chloro-4-nitrophenolate (CNP) during the reaction (Figure S1E). The slight color in the control likely results from minor degradation of GlcNAc-CNP during the reaction. The reaction was also monitored by TLC which showed a new fluorescent spot under UV light (254 nm), indicative of the generation of a new product. This product was confirmed to be UDP-GlcNAz by MS (Figure S1) and ^31^P-NMR spectroscopy (Figure 3A). The ^13^C- and ^1^H-NMR data of the purified UDP-GlcNAz product were consistent with previously reported data (Chen et al., 2011; Vocadlo et al., 2003; Debets et al., 2020) (Supplementary NMR Data). This method of chemoenzymatic UDP-GlcNAz synthesis avoids stepwise multiple reactions, purification of the intermediates, and the isolation/purification of various enzymes, making it superior compared to standard chemical synthesis or multiple-enzyme-catalyzed processes (Chen et al., 2011; Vocadlo et al., 2003; Debets et al., 2020). Our method enables the easy installation of a bioorthogonal handle in UDP-GlcNAc, which is involved in multiple biosynthesis pathways and post-translational modifications in a multitude of organisms (Debets et al., 2020), making it easier to study these processes.

### Synthesis of azido Lipid II and subsequent fluorescent labeling

The two most common variants of Lipid II differ at the third position in the pentapeptide. This residue is L-lysine in the Lipid II of most Gram-positive bacteria, and *m-*DAP in almost all Gram-negative bacteria. So far, in most fluorescently labeled Lipid II variants used in PG-synthesis assays, the fluorescent group was attached to the lysine at the third position of the pentapeptide (Mohammadi et al., 2011, 2014; Breukink et al., 2003; Egan and Vollmer, 2016). This lysine is either directly used as an acceptor in PBP-catalyzed transpeptidation reactions or carries a branch made of L-amino acids that are used in cross-link formation. Hence, the presence of a fluorescent group at the lysine prevents these Lipid II variants from participating in transpeptidase reactions. To be able to measure the cross-linking in PG-synthesis assays, fluorescently labeled lysine Lipid II needs to be mixed with unlabeled Lipid II. We reasoned that Lipid II with a fluorescent label at the GlcNAc could circumvent this need for unlabeled Lipid II. Although MurG can convert Lipid I to Lipid II by utilizing UDP-GlcNAc in the positive control experiments, we failed to synthesize GlcNAz-labeled Lipid II from Lipid I by using purified MurG from *E. coli* under similar conditions (data not shown), indicating that this reaction may require different substrate concentrations or other conditions for optimal conversion to Lipid II. However, we could successfully synthesize GlcNAz-labeled Lys- or *m-*DAP-Lipid II by the reaction of UDP-GlcNAz and the corresponding type of Lipid I in a reaction catalyzed by a *Micrococcus flavus* membrane preparation (data not shown for Lys-Lipid II) (Figures S2A and S2B). Next, we labeled the purified GlcNAz-labeled Lipid II (DAP) after CuAAC conjugation with dansyl alkyne. After further purification (Figure S2D), mass spectroscopy confirmed the presence of the correct product with a mass at *m*/*z* 2246.24 Da ([M−H]^-^) (Figures 3B and S2C). This method provides an efficient way of labeling Lipid II at GlcNAc and complements earlier Lipid II labeling methods that reported the labeling of the pentapeptides or MurNAc residues of Lipid II (Kuru et al., 2012, 2015; Bisson-Filho et al., 2017; Monteiro et al., 2015, 2018; Hsu et al., 2017; Hugonnet et al., 2016; Montón Silva et al., 2018; Breukink et al., 2003; Hasper et al., 2004, 2006; van Dam et al., 2007, 2009; Olrichs et al., 2011; DeMeester et al., 2018; Taylor et al., 2020).

### PG synthesis with dansyl-GlcNAz-Lipid II

**Figure 4:**
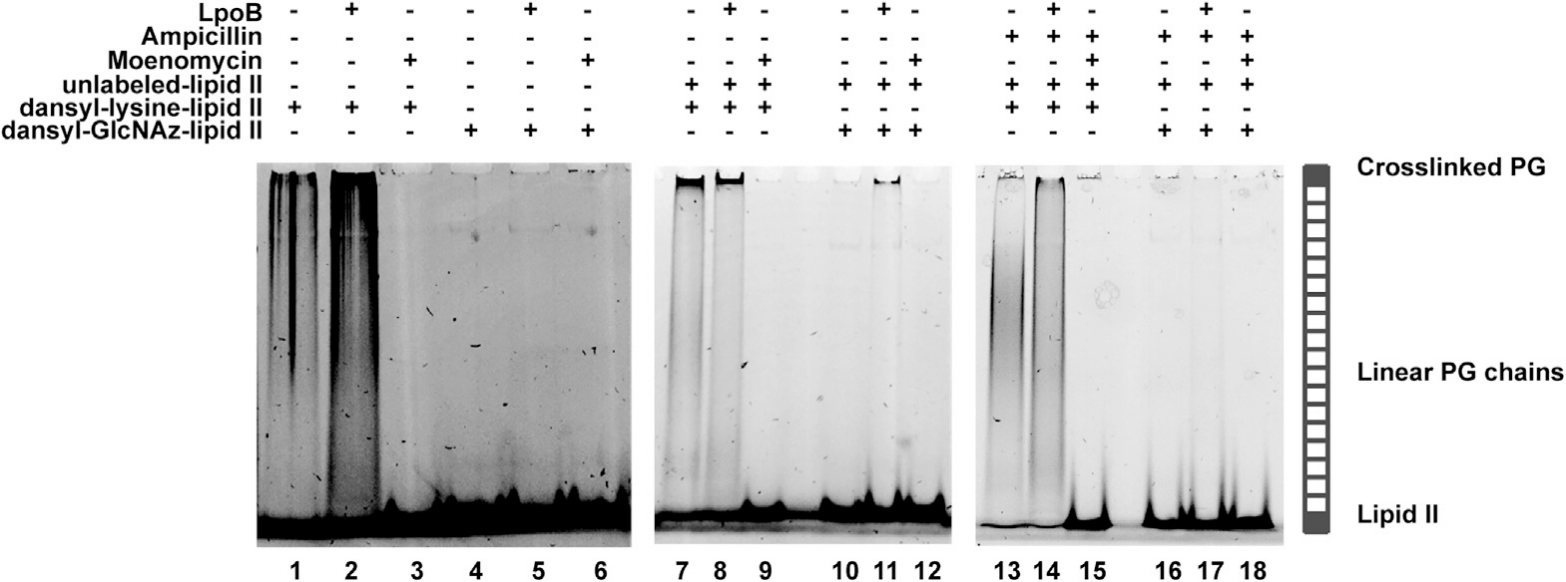
Dansyl-labeled Lipid II versions labeled either at lysine or GlcNAc as substrates for *E. coli* PBP1B.SDS-PAGE analysis of PG products by PBP1B^Ec^ reactions with dansyl-lysine-Lipid II or dansyl-GlcNAz-Lipid II, in the absence of unlabeled Lipid II (lanes 1–6) or presence of unlabeled Lipid II (lanes 7–12 and lanes 13–18) Samples were incubated for 1 h at 37 °C and boiled and subject to SDS-PAGE analysis with fluorescence detection glycan strands were produced from dansyl-lysine-Lipid II (lanes one and 7). In the presence of LpoB, the production of glycan strands of PG increased (lanes 2 and 8). Synthesis of PG was abolished by moenomycin (lanes 3, 9, and 15); the transpeptidation reaction was abolished by the addition of ampicillin (lanes 13 and 14). In reactions with of dansyl-GlcNAz-Lipid II, no production of glycan strands was observed in the absence of unlabeled Lipid II (lanes 4 and 10). In the presence of unlabeled Lipid II and LpoB (lane 11), a small amount of cross-linked-labeled PG was produced, and this product was abolished by the addition of ampicillin (lane 17).

We next tested whether dansyl-GlcNAz-Lipid II was accepted by three class A PBPs from Gram-negative bacteria as a substrate in PG synthesis reactions (Figure S3A). Reactions were carried out in the presence or absence of the respective outer membrane lipoprotein activators. Consumption of Lipid II and formation of glycan strands were monitored by SDS-PAGE (Hernández-Rocamora et al., 2021) (Figures 4 and S3). First, we tested *E. coli* PBP1B (PBP1B^Ec^), which interacts with proteins from the cell division machinery and is a major contributor to the synthesis of septal PG (Egan et al., 2020) (Figure 4). PBP1B^Ec^ was unable to polymerize dansyl-GlcNAz-Lipid II (Figure 4 lane 4), even in the presence of its cognate outer membrane-anchored lipoprotein activator LpoB (Figure 4 lane 5). By contrast, PBP1B^Ec^ polymerized Lipid II labeled with dansyl at the lysine of the pentapeptide (dansyl-lysine-Lipid II), as expected (Figure 4 lane 1). Addition of LpoB increased the production of glycan strands (Figure 4 lane 2) while the GTase-inhibitor moenomycin completely blocked the reaction (Figure 4 lane 3).

One possible explanation for the inability of PBP1B to polymerize dansyl-GlcNAz-Lipid II was that this substrate is too bulky to simultaneously occupy the donor and acceptor sites at the GTase domain, as previously reported for Atto550- and Atto647-modified Lipid II variants (Van’t Veer et al., 2016). Therefore, we added an equal amount of unlabeled Lipid II to reactions to test if it allows reactions to proceed. We observed polymerized glycan strands at the front of the lane when using dansyl-GlcNAz-Lipid II (Figure 4 lane 11) albeit only in the presence of LpoB and the signal was lower than that in the positive control using dansyl-lysine-Lipid II (Figure 4 lanes 7 and 8). To check whether dansyl-GlcNAz-Lipid II was incorporated by the GTase or TPase activities of PBP1B, we tested the effect of the TPase inhibitor ampicillin. Ampicillin blocked the incorporation of dansyl-GlcNAz-Lipid II (Figure 4 lane 17) but could not abolish the incorporation of dansyl-lysine-Lipid II (Figure 4 lane 13 and lane 14). This indicates that the dansyl-GlcNAz-Lipid II was incorporated into PG by the transpeptidase activity of PBP1B, likely used as a donor with the growing chain being used as an acceptor. The amount of incorporation of dansyl-GlcNAz-Lipid II into PG was relatively low, which is in line with the low level of transpeptidase-mediated incorporation of unnatural D-amino acids of 0.2%–2.8% (Hugonnet et al., 2016). Similar results were obtained for a PBP1B homolog from *Pseudomonas aeruginosa* (PBP1B^Pa^) (Figure S3B). We also tested *E. coli* PBP1A and could not find any evidence of GTase or TPase activity with this PBP with dansyl-GlcNAz-Lipid II (Figure S3C). This indicates that the substrate specificity of the TPase activity of PBP1A differs from that of the other two tested PBPs.

### *In vivo* labeling of the PG layer by supplying GlcNAz-CNP to OleD-expressing *E. coli* cells

**Figure 5:**
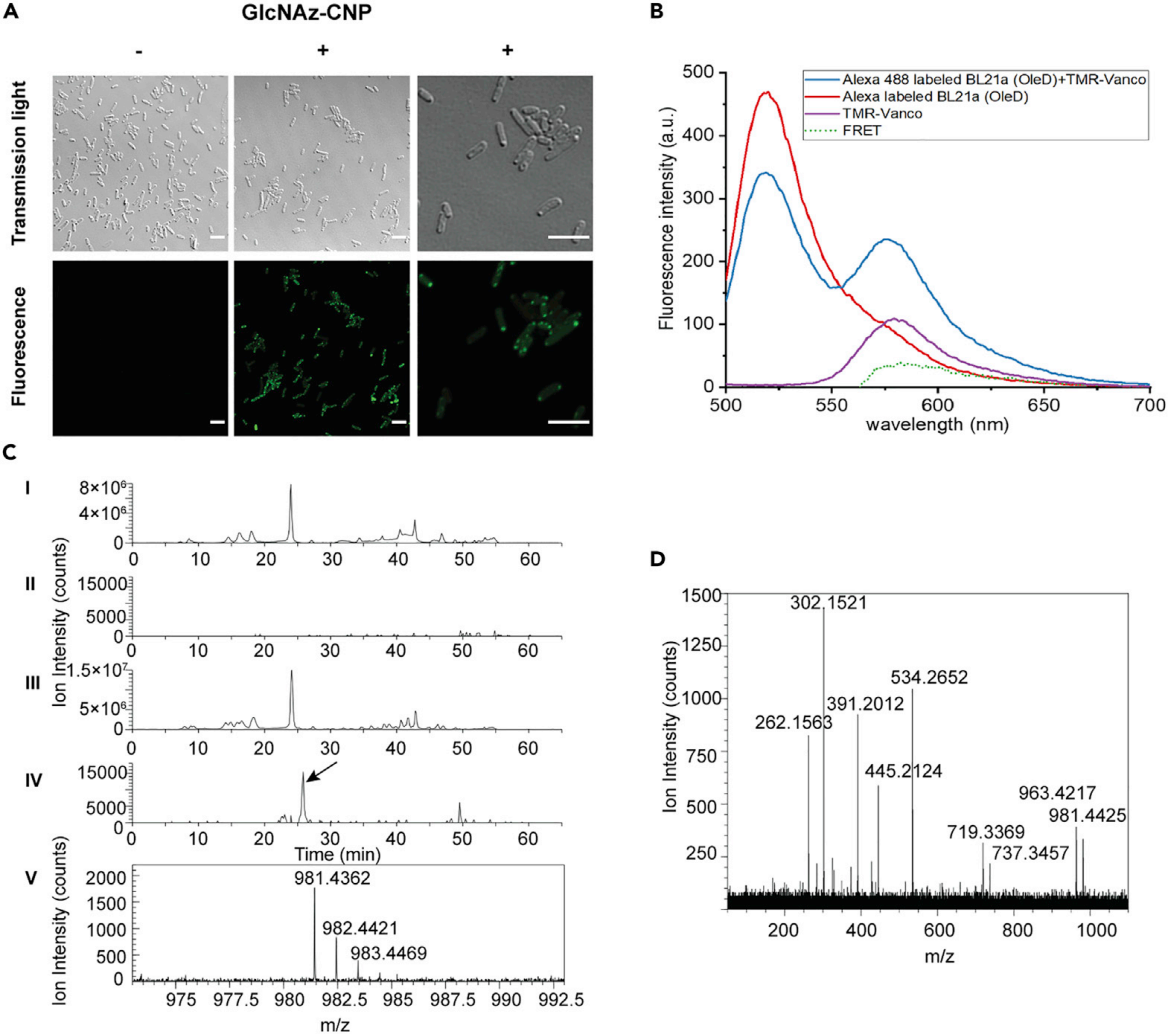
Analysis of *in vivo* labeling of the PG layer of OleD-expressing *E. coli* cells (A) Visualization of GlcNAz incorporation via fluorescent labeling. GlcNAz-labeled bacteria were fixed and incubated with Alexa Fluor 488 alkyne in the presence of Cu(I) for 30 min at room temperature. Cells were washed three times with PBS and transferred onto microscope slides. Transmitted light (top row) and fluorescence (bottom row) images of bacteria grown in LB only (left) or in LB containing GlcNAz-CNP (center and right) are shown. Note that the images on the right have a higher magnification than the other images. Scale bars: 5 μm. (B) The interaction between TMR-vancomycin and Alexa Fluor 488-labeled bacteria leads to FRET. GlcNAz-labeled bacteria were incubated with EDTA to disrupt the outer membrane and allow TMR-Vancomycin access PG. Permeabilized bacteria were then incubated with TMR-Vancomycin (0.8 μM). The red curve represents the fluorescence of Alexa Fluor 488-labeled cells in 1 mL of PBS; the purple curve represents the fluorescence of only TMR-Vancomycin (0.8 μM); the blue curve represents the fluorescence of Alexa Fluor 488-labeled cells treated with TMR-Vancomycin (0.8 μM); the green dashed curve represents the FRET signal (the purple curve subtracted from the blue curve and red curve). (C) Confirmation of GlcNAz incorporation into *E. coli* PG. Traces Ⅰ and Ⅱ show total the ion chromatogram and the mass extracted ion chromatogram for GlcNAz-MurNAc-tetrapeptide, respectively, of the muropeptides isolated from control cells grown in the absence of GlcNAz. Traces Ⅲ and Ⅳ show the corresponding chromatograms of cells grown in the presence of GlcNAz. Trace Ⅴ zooms into the mass spectrum at 25.9 min of GlcNAz-MurNAc-tetrapeptide in the labeled sample indicated by an arrow in Ⅳ. (D) Fragmentation spectrum of the GlcNAz-MurNAc-tetrapeptide ion (*m*/*z* = 981.436) eluting at 25.9 min in trace Ⅳ in C.

We next tested if we could incorporate GlcNAz into PG *in situ* by providing GlcNAz-CNP to *E. coli* cells expressing OleD. We hypothesized that GlcNAz-CNP is converted to UDP-GlcNAz by OleD, using the endogenous pool of UDP. UDP-GlcNAz can then be used to synthesize Lipid II, which in turn will be used for the synthesis of PG. To test this hypothesis, we expressed OleD in BL21(DE3) cells and added GlcNAz-CNP to the growth medium. In principle, the outer membrane of *E.coli* is permeable to a range of small molecules and sugars (Williams et al., 2011), thus it should be possible for BL21 cells to take up GlcNAz-CNP. Indeed, we observed that the color of the cell suspension turned more yellowish after incubation with cells expressing OleD (Figure S4A), while this did not occur with control cells not expressing OleD. This indicates that 2-chloro-4-nitrophenol has been produced as a byproduct of the generation of UDP-GlcNAz inside the cells. Subsequently, the cells were subjected to copper (I)-catalyzed click reaction using Alexa Fluor 488-alkyne to test for the presence of accessible GlcNAz groups. Cells grown in the presence of GlcNAz-CNP were labeled, while cells grown in the absence of GlcNAz-CNP were not labeled (Figure 5A). Thus, this suggests that i) UDP-GlcNAz was produced inside the cells, ii) it is utilized by biosynthesis pathways, and iii) that at least some of the GlcNAz molecules were exported (to the periplasm) and subsequently labeled by the click reaction. The fluorescence appeared in patches on the cells, but also at other locations along the cell periphery. This pattern may point to PG-specific labeling. To test this possibility, we performed a Förster resonance energy transfer (FRET) experiment using fluorescently labeled vancomycin (Mohammadi et al., 2011). We reasoned that if the PG layer was fluorescently labeled with Alexa Fluor 488 attached to the incorporated GlcNAz, then binding of TMR-vancomycin to a nearby D-Ala-D-Ala should lead to FRET. To enable TMR-vancomycin to cross the outer membrane, we utilized EDTA to increase the permeability of the outer membrane of bacteria (MacNair and Brown, 2020). The occurrence of FRET is generally reflected by a decrease in fluorescence of the donor (Alexa Fluor 488) and an increase in fluorescence of the acceptor (TMR-vancomycin). Indeed, adding TMR-vancomycin to the Alexa Fluor 488-labeled cells lead to an FRET signal, indicating that at least part of the UDP-GlcNAz was used for the synthesis of Lipid II and nascent peptidoglycan which were then subsequently labeled (Figure 5B).

Next, we aimed to obtain more direct evidence for the presence of GlcNAz in PG. For this, we isolated PG from cells expressing OleD and grown in the presence of GlcNAz-CNP. We digested the PG with the muramidase cellosyl to produce the disaccharide peptide subunits (muropeptides) and analyzed the muropeptides with nano-LC coupled to a mass spectrometer (Desmarais et al., 2014, 2015). We were able to identify and confirm the presence of the GlcNAz-modified disaccharide tetrapeptide with a determined neutral mass of *m*/*z* = 981.436 (calculated *m*/*z* = 981.401, Figure 5C trace **Ⅳ** and **V**) that was not present in control digestions (Figure 5C trace **Ⅱ**). This proves the presence of GlcNAz in the isolated PG. MS/MS analyses of this molecule supported the identity of the GlcNAz-labeled disaccharide tetrapeptide monomer (Figsure 5D, S4B, and S4C) as the loss of GlcNAz from the parent ion results in an *m*/*z* = 737.346 or, with an extra neutral loss of water, an *m*/*z* = 719.337. Together, these results confirm that GlcNAz was specifically incorporated into bacterial PG. GlcNAz-CNP therefore can be converted into UDP-GlcNAz by heterologous OleD and the resulting precursor is used in the biosynthesis of PG. In our experiments, we did not observe azido MurNAc-containing compounds even though UDP-GlcNAc is a precursor of UDP-MurNAc in the bacterial cell. Most likely, the specificity of enzymes required for the UDP-MurNAc synthesis may be too high and cannot use UDP-GlcNAz as a substrate. This labeling method could likely be applicable to other bacteria, provided that OleD can be heterologously expressed.

By expressing the heterologous glycosyltransferase OleD in bacteria, we could incorporate the unnatural amino sugar GlcNAz into the PG layer. Given the promiscuity of OleD for the sugar nucleotide donor (Gantt et al., 2011, 2013; Williams et al., 2007), this labeling approach may find use in the modification of other sugars implicated in other biosynthetic processes. As we demonstrated here for PG, we envision that this method will be also applicable to introduce GlcNAc modified with a chemical handle or other sugar analogs in a range of hosts. Sugars such as glucose and galactose decorated with unique functionalities such as fluorophores, fluorine, or isotopes may be used this way for subsequent analysis by fluorescent microscopy, NMR, or MS.

### Limitations of the study

Our study is limited to labeling bacteria (or other microorganisms) that express the OleD enzyme or homologs thereof. Lipid II labeled with a fluorophore at the N-acetylglucosamine cannot be used to study the glycosyl-transferase activity of PBPs.

## STAR★Methods

### Key resources table

**Table:** 

REAGENT or RESOURCE	SOURCE	IDENTIFIER
**Bacterial and virus strains**		

*E. coli* Rosetta BL21(DE3) pLysS with pET28a-OleD-TDP16 plasmid	Utrecht Institute for Pharmaceutical Sciences, Utrecht University	N/A
*Micrococcus flavus DSM 1790*	MBB group, Utrecht University	N/A
*Staphylococcus simulans 22*	MBB group, Utrecht University	N/A

**Chemicals, peptides, and recombinant proteins**		

silica	Merck	109385
kanamycin sulfate	Merck	420311
sodium ascorbate	Sigma-Aldrich	11140
IPTG	Thermo Fisher	R0392
imidazole	Sigma-Aldrich	I2399
RNAse B	Sigma-Aldrich	R7884
DNase	Sigma-Aldrich	DN25
lysozyme	Sigma-Aldrich	62971
*E. coli* PBP1B	Centre for Bacterial Cell Biology, Newcastle University	N/A
*P. aeruginosa* PBP1B	Centre for Bacterial Cell Biology, Newcastle University	N/A
*E. coli* PBP1A	Centre for Bacterial Cell Biology, Newcastle University	N/A
*E. coli* LpoB	Centre for Bacterial Cell Biology, Newcastle University	N/A
*P. aeruginosa* LpoP	Centre for Bacterial Cell Biology, Newcastle University	N/A
*E. coli* LpoA	Centre for Bacterial Cell Biology, Newcastle University	N/A
GlcNAz-CNP	This paper	N/A
Triton X-100	Sigma-Aldrich	X100
dansyl alkyne	MBB group, Utrecht University	N/A
undecaprenyl phosphate	MBB group, Utrecht University	N/A
UDP-GlcNAz	This paper	N/A
TMR-Vancomycin	MBB group, Utrecht University	N/A
ammonium bicarbonate	Sigma-Aldrich	A6141
Alexa Fluor™ 488 Alkyne	Thermo Fisher	A10267
ammonium hydroxide	Sigma-Aldrich	221228
tris-hydroxypropyltriazolylmethylamine	Sigma-Aldrich	762342
Glucosamine hydrochloric acid salt	Sigma-Aldrich	G4875
chloroacetic anhydride	Sigma-Aldrich	215163
2-chloro-4-nitrophenol	Sigma-Aldrich	C61208
Sodium azide	Sigma-Aldrich	S2002

**Software and algorithms**		

Prism 8	GraphPad	https://www.graphpad.com/
MestReNova	Mestrelab	https://mestrelab.com/
Image J 1.53c	National institutes of Health	https://imagej.net/software/fiji/
Adobe Illustrator	Adobe Inc.	https://www.adobe.com/products/illustrator.html
Xcalibur™ Software	Thermo Scientific™	https://www.thermofisher.com/order/catalog/product/OPTON-30965
Excel	Microsoft	https://www.office.com/
ChemDraw16.0	PerkinElmer	https://perkinelmerinformatics.com/products/research/chemdraw/

**Other**		

Ni-NTA Agarose	QIAGEN	30210
PD-10 desalting column	GE Healthcare	GE17-0851-01
Amicon® Ultra-15 Centrifugal Filter Ultracel-30	Merck Millipore Ltd.	UFC903008

### Resource availability

#### Lead contact

All requests for reagents and resources should be directed to the lead contact, Eefjan Breukink (E.J.Breukink@uu.nl).

#### Materials availability

Synthetic routes to all chemical compounds are described in Supplemental Information. Where available, these may be shared by the lead contact. Enzymes, reagents, and bacteria used for the biological studies were obtained from the commercial or internal sources described in the key resources table.

### Experimental model and subject details

*E. coli* Rosetta BL21(DE3) pLysS transformed with pET28a-OleD-TDP16 plasmid was used for the production of OleD and the *in vivo* incorporation of GlcNAz into the peptidoglycan layer. Experiments were conducted at 37°C unless otherwise noted. All information on alternate temperature conditions, as well as media conditions for experiments, can be found in the method details section.

### Method details

#### Materials and instrumentation

All reagents employed were of American Chemical Society (ACS) grade or finer and were used without further purification unless otherwise stated. The compounds were purified by flash silica column chromatography (Merck, silica gel 60, 230∼400 mesh). All reactions and fractions from column chromatography were monitored by thin-layer chromatography (TLC) using plates with or without a UV fluorescent indicator (Merck 60 F_254_, SO_2_). Iodine (Merck) staining also was used for visualization of TLC. UDP-MurNAc-pentapeptide extract was obtained according to the previously reported method (Kohlrausch and Höltje, 1991). Undecaprenyl-phosphate was isolated from bay leaves and phosphorylated as described (Swiezewska et al., 1994; Danilov et al., 1989). Dansyl-lysine-lipid II was prepared as published (Breukink et al., 2003; Schwartz et al., 2002).

All NMR spectra were recorded at 400 MHz in Utrecht Institute for Pharmaceutical Sciences center with chemical shifts reported in parts per million (ppm) downfield relative to tetramethylsilane (TMS). ^1^H NMR data are reported in the following order: multiplicity (s, singlet; d, doublet; t, triplet; q, quartet; and m, multiplet), number of protons, and coupling constants (J) in Hertz (Hz). When appropriate, the multiplicity is preceded by br, indicating that the signal was broad. ^13^C NMR spectra were recorded at 100 or 125 MHz with chemical shifts reported relative to CDCl_3_ δ 77.0. High-resolution mass spectrometry (HRMS) analysis was performed using an HRMS ESI-TOF or ESI-MS (Thermo Finnigan, LCQDECA XP) instrument. The fluorescent image of labeled bacteria was taken by Confocal Zeiss LSM 880 FAST AiryScan at the Biology Imaging Center at the Faculty of Science of Utrecht University.

#### Protein production and purification

We utilized a protocol based upon the previously published methods for the OleD enzyme (Gantt et al., 2011). A single colony of *E. coli* Rosetta BL21(DE3) pLysS transformed with pET28a-OleD-TDP16 plasmid, which was picked from the LB agar plate with 50 μg mL^−1^ kanamycin, was inoculated in 60 mL of LB with 50 μg mL^−1^ kanamycin at 37°C and in a shaker (200 rpm) overnight. The next morning, the starting culture was transferred into 6 L of LB medium supplemented with 50 μg mL^−1^ kanamycin and further grown in an incubator with shaking (200 rpm) at 37°C until the OD_600nm_ reached around 0.6. IPTG was added into the medium cultures to bring the final concentration of IPTG to 0.4 mM and the cultures were incubated at 25°C for around 16 h with shaking at 200 rpm. Bacteria were concentrated further by centrifugation at 6000*g* and 4°C for 15 min. The cell pellet was subsequently resuspended in 100 mL of lysis buffer (20 mM phosphate buffer, pH 7.4, 0.5 M NaCl, 10 mM imidazole, 50 μg mL^−1^ RNase, 10 μg mL^−1^ DNase and 0.25 mg mL^−1^ lysozyme) and lysed with a cell disruptor (Constant systems) for 5 cycles while cooling at 4°C. After the lysis, the disrupted bacteria cells were incubated on ice for a further 20 min to degrade peptidoglycan, DNA, and RNA. Cell debris was removed by centrifugation (38,000 *g* at 4°C for 15 min) and then the supernatant was applied to 6 mL of nickel resin equilibrated in wash buffer (20 mM phosphate buffer, pH 7.4, 0.3 M NaCl, 10 mM imidazole). The protein was incubated with the beads for 2 h on a digital tube roller at 4°C. Then the resin was applied to a column followed by a washing step with 150 mL of chilled wash buffer. Subsequently, the protein was eluted with 10 mL of elution buffer (20 mM phosphate buffer, pH 7.4, 0.3 M NaCl, 240 mM imidazole). Purified protein was concentrated and desalted using a PD-10 desalting column (GE Bioscience, USA), equilibrated with 50 mM Tris-HCl (pH 8.0), to provide 3 mL of the desired protein. The purity of the enzyme was determined by 10% SDS-PAGE, indicating the purity of more than 95%. The purified enzyme was stored at −80°C until use.

#### Chemoenzymatic synthesis of UDP-GlcNAz

Reactions were conducted at 37°C in 20 mL Tris-HCl buffer (50 mM, pH 8.0) with GlcNAz-CNP (5 mM), UDP (5 mM), MgSO_4_ (25 mM), and OleD enzyme (125 μM). The reaction was monitored by TLC (2:1:1, n-butanol: acetic acid: H_2_O as eluent, A_254 nm_). Besides, the color of the reaction mixture turned yellowish from almost colorless as the reaction progressed. After 24 h, the reaction was stopped by adding 1 mL chloroform to precipitate the OleD enzyme. The reaction medium was centrifuged at 2,000*g* to remove the OleD enzyme. The aqueous upper phase was carefully collected and transferred into a new tube, followed by lyophilization. The dried yellowish residue was dissolved in 0.25 mL of Milli-Q water and the desired product was purified by silica gel column using *n*-butanol: acetic acid: water (2:1:1) as eluent. The product (UDP-GlcNAz, R_f_ = 0.28, *n*-butanol: acetic acid: water = 2:1:1) was concentrated on a vacuum rotary evaporator and the residue was lyophilized to get a white powder (39 mg, yield 61%).

#### NMR, and MS spectra of UDP-GlcNAz

^1^H NMR (400 MHz, D_2_O) δ 7.81 (d, *J* = 8.1 Hz, 1H), 5.94 – 5.73 (m, 2H), 5.52 – 5.31 (m, 1H), 4.27 – 4.19 (m, 2H), 4.16 – 3.89 (m, 7H), 3.74 (ddd, J = 24.3, 16.1, 9.2 Hz, 4H), 3.52 – 3.38 (m, 2H), 3.24 – 3.19 (m, 1H).

^13^C NMR (101 MHz, D_2_O) δ 170.85, 166.40, 151.88,141.56, 102.56, 94.26, 88.60, 82.96 (d, *J* = 8.9 Hz),73.65, 72.99, 70.70, 69.45 (d, *J* = 14.3 Hz), 65.01, 60.24, 53.63 (d, *J* = 8.7 Hz), 51.52, 50,45.

^31^P NMR (162 MHz, D_2_O) δ −11.44 (d, *J* = 20.4 Hz), −13.20 (d, *J* = 21.0 Hz).

HRMS (ESI-TOF) m/z calcd for C_17_H_25_N_6_O_17_P_2_ (M-H) 647.0928, found 647.0933.

#### Isolation of membranes from *Micrococcus flavus/Staphylococcus simulans* (Konings et al., 1973)

A single colony of *Micrococcus flavus DSM 1790* or *Staphylococcus simulans 22* bacteria was inoculated in 6 × 25 mL of CASO broth and grown overnight at 37°C in a shaker. The next morning, the culture was transferred into 4 L of fresh CASO medium, grown until an OD_600nm_ = 1.0∼1.2 was reached, and harvested by centrifugation at 3,800 *g* at 4°C for 30 min. The supernatants were discarded, and bacteria were washed in Tris-HCl (50 mM, pH 7.5, 67 mL of buffer per pellet obtained from one-liter culture). The bacteria were resuspended in 50 mL per gram of wet weight in Tris-HCl buffer (50 mM, pH 7.5). A freshly prepared solution of DNAse and RNAse (both at 0.6 mg per gram of wet weight) in Milli-Q water was added as well as a solution of lysozyme in Milli-Q water (20 mg per gram of wet weight). The cells were then disrupted by 10 cycles in a cell disruptor (2.5 kPa). The resulting suspension was centrifuged at 4,000*g* and the supernatant was collected. The membrane fraction was obtained by centrifugation of the supernatant at 45,000*g*. The membranes were resuspended into 5 mL of Tris-HCl buffer (50 mM, pH 7.5) and frozen at −20°C until further use.

#### The synthesis of GlcNAz-Lipid II from UDP-GlcNAz

30 μL of a 1 mM undecaprenyl phosphate (11-*P*) stock solution in acetone was dried under a nitrogen flow and mixed with 50 μL of UDP-GlcNAz (3 mM), 5 μL of Triton X-100 (10%), 30 μL of the membrane isolation, 5 μL of MgCl_2_ (0.15 M), 15 μL of Tris-Cl (1 M, pH 8.0), and 30 μL of UDP-MurNAc-pentapeptide (UDP-Mpp) extract. The mixture was incubated at room temperature overnight. The reaction was stopped by adding 180 μL of butanol/6 M pyridine-acetate acid (2:1, v/v, pH 4.2). The sample was thoroughly mixed followed by brief centrifugation to separate the different layers. The butanol layer was collected, washed with 100 μL of water, diluted with 400 μL of methanol, and loaded onto a DEAE-cellulose column (diameter: 5 mm, length: 8 mm). The column was rinsed with methanol (600 μL) and eluted with 0 mM, 100 mM, 300 mM, and 500 mM NH_4_HCO_3_ in the water fraction of chloroform/methanol/water (2:3:1), 600 μL for each concentration of eluent. 100 μL of each fraction was collected, dried, and dissolved in chloroform/methanol (2:1), analyzed by TLC with an eluent of chloroform/methanol/water/ammonium hydroxide (88:48:10:1), and stained by iodine. The fractions containing GlcNAz-Lipid II were lyophilized to give 75% yield.

#### Click chemistry and synthesis of the dansyl-GlcNAz-Lipid II

CuSO_4_ (150 μL, 1 mM) and tris-hydroxypropyltriazolylmethylamine (THPTA) (Sigma-Aldrich) (375 μL, 1.5 mM) were mixed in Tris-HCl buffer (10 mM, pH 8.0) and stirred at room temperature for 20 min. Sodium ascorbate (7.5 μL, 500 mM) was added to the reaction mixture and the mixture was immediately dried under nitrogen flow. Subsequently, the residue was dissolved in Tris-HCl buffer (1395 μL, 10 mM, pH 8.0) with 0.1% Triton X-100 and transferred into a tube containing a premixed solution of GlcNAz-labeled Lipid II (100 μL, 1.5 mM) and dansyl alkene (4.5 μL, 100 mM) to bring final concentrations of CuSO_4_ (0.1 mM), THPTA (0.5 mM), sodium ascorbate (2.5 mM), GlcNAz-DAP-Lipid II (0.1 mM) and dansyl alkyne (0.3 mM). The total volume of the reaction is 1.5 mL. The reaction was protected from the light and stirred at room temperature for 2 h, followed by extraction of the Lipid II by 1.8 mL of butanol/6 M pyridine-acetate (2:1, v/v, pH 4.2). The butanol layer was washed with 1 mL water, collected, diluted with 4 volumes of methanol, and loaded onto a DEAE-Cellulose column (diameter: 5 mm, length: 8 mm). The column was, rinsed with 6 volumes of methanol, and eluted with 5 mL buffers at each step solvent gradient of chloroform/methanol/water (2:3:1), chloroform/methanol/100 mM ammonium bicarbonate (2:3:1), chloroform/methanol/200 mM ammonium bicarbonate (2:3:1), chloroform/methanol/300 mM ammonium bicarbonate (2:3:1), chloroform/methanol/500 mM ammonium bicarbonate (2:3:1), and to chloroform/methanol/1 M ammonium bicarbonate (2:3:1). The dansyl-Lipid II was present in the fraction of chloroform/methanol/1 M ammonium bicarbonate (2:3:1). The final product was obtained after lyophilizing the fractions (yield, 93%). The neutral mass of dansyl-GlcNAz-Lipid II was determined as 2247.24 Da which is consistent with the calculated exact mass of 2246.15 Da.

#### PG synthesis assay using class A penicillin-binding proteins

*E. coli* PBP1B, *P. aeruginosa* PBP1B, *E. coli* PBP1A, *E. coli* LpoB, *P. aeruginosa* LpoP, and *E. coli* LpoA were purified as previously described (references: E.c. PBP1B (Bertsche et al., 2006), P.a. PBP1B (Caveney et al., 2020), PBP1A, E.c. LpoB (Born et al., 2006), P.a. LpoP (Egan et al., 2014), E.c. LpoA (Jean et al., 2014)). Reactions were started by mixing class A PBPs (0.5 μM for PBP1B^Ec^ and PBP1B^Pa^ or 0.7 μM for PBP1A^Ec^) in the presence or absence of activators (2 μM for LpoB and LpoP or 2.8 μM for LpoA) with 10 μM labeled substrate (dansyl-GlcNAz-Lipid II or dansyl-lysine-Lipid II) in 50 mM Hepes at pH 7.5, 150 mM NaCl, 10 mM MgCl_2_, 0.05% TX-100. When indicated, reactions were supplemented with 10 μM unlabeled *m*-DAP-Lipid II, 50 μM moenomycin or 1 mM ampicillin. Reactions were incubated for 1 h (PBP1B^Ec^ and PBP1B^Pa^) or 2 h (PBP1A^Ec^) at 37 °C and then boiled for 5 min. Samples were vacuum-dried using a speed-vac desiccator and products were analyzed by Tris-Tricine SDS-PAGE as described previously (Hernández-Rocamora et al., 2021), with the exception that gels were visualized using a UV transilluminator.

#### Test for GlcNAz incorporation by intact bacteria

Overnight pre-cultured BL21 cells harboring pET28a-OleD-TDP16 were inoculated into fresh LB medium (with 50 μg mL^−1^ kanamycin) and were incubated until the OD_600nm_ was about 0.6. 1 mL of cells were collected by centrifugation at 6,000*g* for 2 min BL21 (OleD) were resuspended in 200 μL of LB medium (with 50 μg mL^−1^ kanamycin). GlcNAz-CNP (0.2%) and 0.4 mM IPTG were added to the cell samples. Cells were incubated at 37°C for 2 h and then collected (6,000*g*, 2 min) and washed twice with 400 μL PBS buffer (pH 7.4). Subsequently, the cells were resuspended in PBS (pH 7.4) buffer and fixed at room temperature with 4% paraformaldehyde in PBS (pH 7.4) for 20 min. The cells were then washed twice with 200 μL PBS (pH 7.4). The cells were collected and redispersed in 25 μL of isopropanol: water (2:1) followed by the addition of 5 μL of CuSO_4_ solution (10 mM), 5 μL of THPTA (Sigma-Aldrich) (10 mM), 5 μL of freshly prepared sodium ascorbate (Sigma-Aldrich) (25 mM), and 10 μL of Alexa Fluor 488-alkyne (Sigma Aldrich) (100 μM). Cells were incubated at room temperature in a shaker for 30 min in the dark, subsequently washed 6 times with 200 μL of PBS (pH 7.4) and resuspended in 200 μL of PBS (pH 7.4) then prepared for imaging.

#### FRET experiment of the Alexa fluor 488 labeled BL21 (pET28a-OleD-TDP16) and TMR-vancomycin

BL21 (pET28a-OleD-TDP16) cells were grown in LB with GlcNAz-CNP (0.2%) and labeled with Alexa fluor 488 via click chemistry. The labeled bacteria were resuspended into 1 mL of PBS (OD_600nm_ = 0.06) and EDTA was added into the suspension to bring the concentration to 1.7 mM. The bacteria were incubated with EDTA for 20 min at room temperature. Afterward, the bacterial suspension was scanned by a fluorometer (excited at 488 nm and data recorded between 500 nm and 700 nm). Then, TMR-Vancomycin (0.8 μM) was added to the suspension that was incubated for 20 min at room temperature. The data of the emission spectrum was recorded between 500 nm and 700 nm. The data of the control group (0.8 μM TMR-vancomycin in PBS with 1.7 mM EDTA) was recorded at the same conditions and fluorometer settings as the other sample. The FRET signal was plotted by the subtraction of the emission spectrum of TMR-Vancomycin from the emission spectrum of the mixture of Alexa fluor 488 labeled BL21 (pET28a-OleD-TDP16) and TMR-Vancomycin, which was recorded between 500 nm and 700 nm and excited at 488 nm.

#### PG preparation and analysis

BL21 pET28a-OleD-TDP16 cells were grown in 100 mL of LB medium in the presence or absence of 0.1% GlcNAz-CNP and the PG was isolated from these cells as previously published (Glauner, 1988). PG was digested overnight at 37°C with cellosyl in 20 mM ammonium formate (pH 4.8) and the samples were boiled for 10 min. The resulting muropeptides were reduced by adding 0.5 M ammonium hydroxide (pH 9.0, adjusted with formic acid) and ∼1 mg of solid tetramethylammonium borohydrate and incubating for 30 min at room temperature. The pH was adjusted to 3-4 with 20% formic acid prior to LC/MS analysis.

LC/MS analysis was performed as recently described (Taylor et al., 2021) with modifications as detailed below. For LC-MS/MS analysis 20 μL of muropeptide digest was acidified by addition of 1/10^th^ volume 10% formic acid (aq) and placed in an autosampler vial. 2 μL of acidified muropeptide was injected onto a microbore RP-HPLC column (ACE Ultracore 2.5 SuperC18, 0.5 × 150 mm) flowing at 15 μL min^−1^ delivered by a NanoAcquity UPLC system (Waters, UK). The column temperature was 35°C. Buffer A was composed of water containing 0.1% (v/v) formic acid. Buffer B was acetonitrile containing 0.1% (v/v) formic acid. The following elution gradient was used: starting at 0% buffer B, rising to 1.3% B at 3 min, then on to 2.5% B at 20 min, rising to 6.0% B at 35 min, the gradient was ramped to 10% B at 45 min, then to 50% B at 50 min, followed by 2 min at 85% B, and finally 12.5 min re-equilibration at 0% B. The total run time was 65 min.

The HPLC column eluate was directed to the mass spectrometer (Impact II QTof, Bruker) via an Apollo electrospray ion source (Bruker). The settings for the ion source were: capillary voltage and temperature settings of 3,200 V and 150 °C respectively, together with a dry gas flow of 6 L min^−1^ and nebulizer pressure of 1.0 bar. MS data were acquired in positive ion mode over the range of 50∼2,000 m/z at a spectral rate of 2 Hz. MS/MS acquisition was performed on the top five intense precursors in each scan (ions excluded after two MS/MS events). The preferred charge state range was set at +1 to +4 (undetermined charge states were excluded) and the sampling rate varied from 0.5 Hz at low ion counts (10,000) to 5.0 Hz for high-intensity ion counts (500,000). The resulting MS spectral data was opened for analysis using Compass DataAnalysis^TM^ software (Bruker).

#### The synthesis of GlcNAz-CNP and NMR data

##### Synthesis of compound 1

Glucosamine hydrochloric acid salt (3.0 g, 13.92 mmol, 1 eq) was dissolved in methanol (150 mL). Sodium methoxide (3.09 mL; 1 eq, 25% w/w, 4.5 M, in Methanol) was added into the solution drop by drop. The solution was stirred at room temperature for 20 min. Triethylamine (1.94 mL, 13.92 mmol, 1 eq) and chloroacetic anhydride (2.61 g, 15.3 mmol, 1.1 eq) were subsequently added to the reaction solution. The reaction was kept stirring for up to 72 h, during which the reaction was monitored by TLC. When the starting material was consumed up, the reaction solution was filtered, and the filter residue was washed with methanol (50 mL). The filtrate was concentrated and dried to offer the residue. To a 250 mL round flask, the residue in acetyl chloride (75 mL) was added. The solution was kept stirring for 20 h at room temperature. The color of the reaction solution got dark from colorless. Then the solution was diluted by chloroform (400 mL), treated with the ice-water mixture (400 mL), washed with cold water (250 mL), and cold saturated sodium bicarbonate solution (3 × 400 mL). The organic layer was dried with magnesium sulfate and concentrated with a vacuum rotary evaporator. The brown residue was purified with silica gel column (230∼400 mesh, Merck corporation) with eluting (EtOAc/Hexane, from 1:5 to 2:1) to afford compound **1** as light-yellow solid (3.54 g, 8.85 mmol, 63.6%).

^1^H NMR (400 MHz, CDCl_3_) δ 6.86 (d, *J* = 8.4 Hz, 1H, *NH*), 6.19 (d, *J* = 3.7 Hz, 1H, *H-1*), 5.38 (dd, *J* = 10.4, 9.7 Hz, 1H, *H-3*), 5.20 (td, *J* = 9.6, 2.6 Hz, 1H, *H-4*), 4.47 (ddd, *J* = 10.7, 8.5, 3.8 Hz, 1H, *H-2*), 4.29 (dd, 1H, *H-6*), 4.27 – 4.25 (m, 1H, *H-5*), 4.11 (dd, 1H, *H-6′*), 4.05 – 3.94 (m, 2H, *H-9),* 2.08 – 2.03 (m, 9H, *CH*_*3*_).

^13^C NMR (101 MHz, CDCl_3_) δ 171.11, 170.47, 169.11 (*C14, C18, C22*), 166.45 (*C8*), 92.71 (*C1*), 70.92 (*C5*), 69.70 (*C3*), 66.84 (*C4*), 61.05 (*C6*), 53.88 (*C2*), 42.06 (*C9*), 20.62, 20.55, 20.49 (*C16, C19, C23*).

##### Synthesis of compound 2

To a 50 mL round flask, compound 1 (100 mg, 0.25 mmol) and acetonitrile (2.5 mL) were added. 2-chloro-4-nitrophenol (86.5 mg, 0.5 mmol, 2 eq) and trimethylamine (2.5 mL) was added subsequently. After the reaction was kept stirring at 40°C overnight, the reaction mixture was diluted with chloroform (10 mL), washed with sodium hydroxide solution (0.1 M) twice (2 × 5 mL), and washed with water (10 mL) before being dried by magnesium sulfate. After filtration, the filtrate was concentrated with a vacuum rotary evaporator and the resulting residue was purified by silica gel (230∼400 mesh, Merck corporation) column chromatography (Hexane/EtOAc, 1:1). Compound **2** was obtained as an off-white solid (95 mg, 0.177mmol, 71.4%).

^1^H NMR (400 MHz, CDCl_3_) δ 8.28 (d, *J* = 2.7 Hz, 1H, *H-18*), 8.11 (dd, *J* = 9.1, 2.7 Hz, 1H, *H-16*), 7.26 (d, *J* = 9.2 Hz, 2H, *H-15*), 6.89 (d, *J* = 8.3 Hz, 1H, *NH*), 5.52 (dd, *J* = 10.5, 8.1 Hz, 1H, *H-1*), 5.51 – 5.49 (m, 1H, *H-3*), 5.15 (t, *J* = 8.9 Hz, 1H, *H-4*), 4.25 (qd, *J* = 12.3, 4.3 Hz, 2H, *H-6*, *H-6′*), 4.18 – 4.10 (m, 1H, *H-2*), 4.01 (d, *J* = 3.4 Hz, 1H, *H-5*), 3.99 – 3.93 (m, 1H, *H-11*), 2.19 – 1.99 (m, 9H, *CH*_*3*_).

^13^C NMR (101 MHz, CDCl_3_) δ 170.36, 170.35, 169.24 (*C-23, C-27, C-31*), 166.72 (*C-10*), 157.18 (*C-14*), 143.11 (*C-17*), 126.12 (*C-18*), 124.83 (*C-19*), 123.54 (*C-16*), 116.64 (*C-15*), 98.36 (*C-1*), 72.59 (*C-5*), 70.25 (*C-3*), 68.01 (*C-4*), 61.92 (*C-6*), 54.53 (*C-1*), 42.36 (*C-11*), 20.62, 20.58 (*C-24, C-28, C-32*).

##### Synthesis of compound 3

The compound **2** (54 mg, 0.1 mmol) was dissolved in dimethylformamide in a 50 mL round flask with a stirring bar. Sodium azide (65 mg, 1.0 mmol, 10 eq) was added to the reaction mixture before the reaction was kept stirring for 6 h at 60°C. The reaction mixture was poured into ice water (20 mL) and extracted with ethyl acetate 3 times (3 × 20 mL). The organic phase was collected and washed with water 3 times (3 × 20 mL). The organic phase was washed with saturated sodium bicarbonate solution (10 mL) before being washed with water 3 times (3 × 20 mL) again and dried with magnesium sulfate. The organic phase was filtered, concentrated and the residue was purified by silica gel (230∼400 mesh, Merck corporation) column chromatography (Hexane/EtOAc, 1:2). Compound **3** was obtained as white solid (49 mg, 0.090 mmol, 90.2%).

^1^H NMR (400 MHz, CDCl_3_) δ 8.25 (d, *J* = 2.7 Hz, 1 H, *H-18*), 8.08 (dd, *J* = 9.1, 2.7 Hz, 1H, *H-16*), 7.25 (d, *J* = 9.1 Hz, 1H, *H-15*), 6.79 (d, *J* = 8.4 Hz, 1H, *NH*), 5.56 – 5.53 (m, 1H, *H-1*), 5.53 – 5.48 (m, 1H, *H-3*), 5.14 (t, *J* = 9.2 Hz, 1H, *H-4*), 4.24 (qd, *J* = 12.3, 4.2 Hz, 2H, *H-6*, *H-6′*), 4.14 (dd, *J* = 18.2, 8.2 Hz, 1H, *H-2*), 4.00 – 3.93 (m, 1H, *H-5*), 3.92 (s, 2H, *H-11*), 2.06 – 2.05 (m, 9H, *CH*_*3*_).

^13^C NMR (101 MHz, CDCl_3_) δ 170.43, 170.36, 169.25 (*C-23, C-27, C-31*), 167.36 (*C-10*), 157.21(*C-14*), 143.08 (*C-17*), 126.12 (*C-18*), 124.75 (*C-19*), 123.56 (*C-16*), 116.55 (*C-15*), 98.43 (*C-1*), 72.55 (*C-5*), 70.58 (*C-3*), 68.06 (*C-4*), 61.90 (*C-6*), 54.48 (*C-2*), 52.59 (*C-11*), 20.67, 20.62, 20.58 (*C-24, C-28, C-32*).

##### Synthesis of compound 4

To compound **3** (1.0 g, 18.4 mmol) in anhydrous methanol (30 mL) was added 25% (w/w) sodium methoxide (460 μL, 1 eq). After the reaction was stirred overnight, the solution turned a little yellowish the next morning. Amberlite IRC-50 resin was washed with methanol and added to the reaction mixture, which was stirred until the pH of the mixture reached 5 to 6. The mixture was filtered by an Amberlite IRC-50 resin desalting column and concentrated, purified by silica gel column chromatography with eluent (MeOH/DCM, from 1:10 to 1:5) to afford the off-white compound 4 (630 mg, 82.9%).

^1^H NMR (400 MHz, DMSO) δ 8.31 – 8.25 (m, 1H, *H-20*), 8.17 (d, *J* = 9.1 Hz, 1H, *H-18*), 7.45 (d, *J* = 9.3 Hz, 1H, *H-17*), 5.20 (d, *J* = 8.5 Hz, 1H, *H-1*), 3.84 (dd, *J* = 17.6, 5.2 Hz, 1H, *H-2*), 3.77 (d, *J* = 5.4 Hz, 2H, *H-11*), 3.72 (d, *J* = 11.1 Hz, 1H, *H-6*), 3.53 – 3.51 (m, 1H, *H-6′*), 3.50 – 3.44 (m, 2H, *H – 3*, *H – 4*), 3.26 (d, *J* = 9.2 Hz, 1H, *H-5*).

^13^C NMR (101 MHz, DMSO) δ 166.58 (*C-10*), 157.06 (*C-16*), 141.12 (*C-19*), 124.70 (*C-20*), 123.56 (*C-18*), 121.89 (*C-21*), 115.44 (*C-17*), 98.49 (*C-1*), 76.90 (*C-3*), 72.73 (*C-4*), 69.13 (*C-5*), 59.71 (*C-6*), 54.37 (*C-2*), 50.35 (*C-11*).

ESI-MS m/z calcd for [C_14_H_16_ClN_5_O_8_Na] ^+^ ([M+Na] ^+^) 440.05, found 440.06.

### Quantification and statistical analysis

The fluorescence intensity in the FRET experiments was measured by Cary Eclipse Fluorescence Spectrometer. The raw data was collected by Cary Eclipse Scan Application 1.1. The experiments were repeated at least twice. The figure was produced with GraphPad Prism version 8.0.2 from the raw data.

## Data Availability

• All data reported in this paper will be shared by the lead contact upon request. • This paper does not report original code. • Any additional information required to reanalyze the data reported in this paper is available from the lead contact upon request.

## Supplemental information

Document S1. Figures S1–S4, Schemes S1–S4 and Data S1–S5

## Footnotes

Supplemental information can be found online at https://doi.org/10.1016/j.isci.2022.104753.
