# Supplementary material for: Metabolic labeling of the bacterial peptidoglycan by functionalized glucosamine
Source: iScience. 2022 Jul 12;25(8):104753. doi: 10.1016/j.isci.2022.104753 (PMC9356107; doi:10.1016/j.isci.2022.104753)

iScience, Volume *25*

Supplemental information

Metabolic labeling of the bacterial

peptidoglycan by functionalized glucosamine

Yang Xu, Víctor M. Hernández-Rocamora, Joseph H. Lorent, Ruud Cox, Xiaoqi Wang, Xue Bao, Marjon Stel, Gaël Vos, Ramon M. van den Bos, Roland J. Pieters, Joe Gray, Waldemar Vollmer, and Eefjan Breukink

Supplemental Figures:

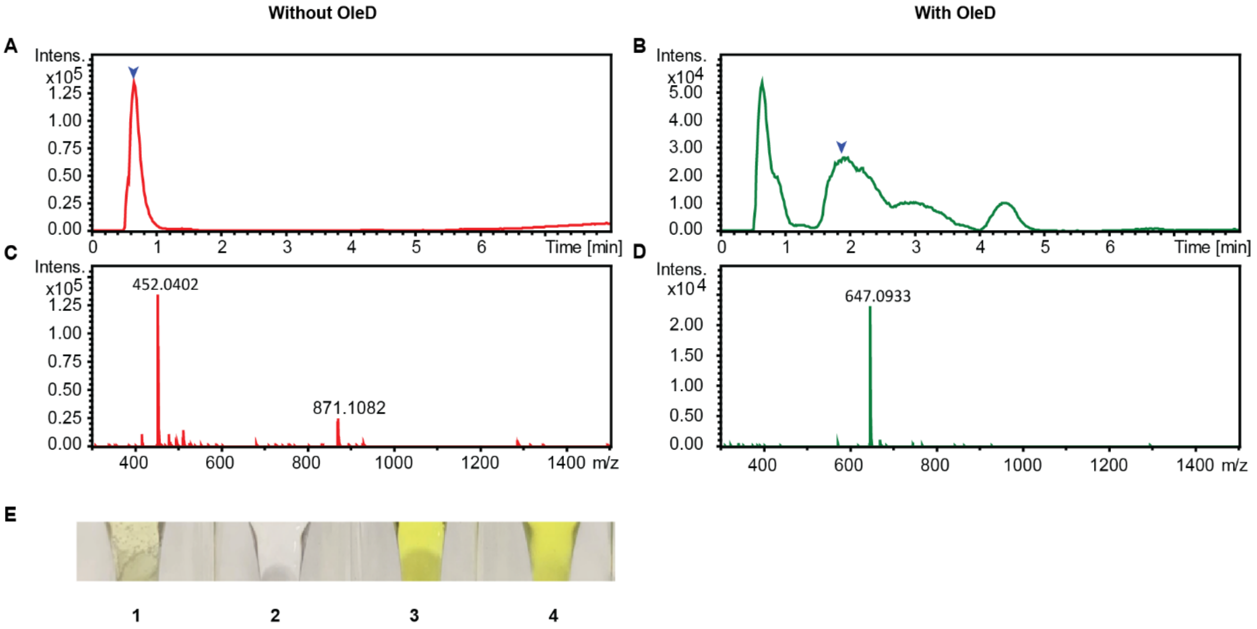

Figure S1. LC-Mass analysis of reactions of GlcNAz-CNP and UDP without (A and C) and with (B and D) OleD and **colorimetric analysis of the reaction, related to Figure 3A. A**) LC-chromatograms with total ion count on the Y-axis of the control group (without OleD); **B**) LC-chromatograms with total ion count on the Y-axis of the experimental group (with OleD); **C**) MS spectra taken at the arrow in **A** showing that GlcNAz-CNP wasn't consumed (452.0402 Da ([GlcNAz-CNP + Cl^-]$^-$)), and **D**) MS spectra taken at the arrow in **B** showing the formation of UDP-GlcNAz (647.0933 Da ([UDP-GlcNAz−H]$^-$)$^-$). Reactions were conducted at 37 °C overnight in 20 mL Tris-HCl buffer (50 mM, pH 8.0) with GlcNAz-CNP (5 mM), UDP (5 mM), $MgSO_4$ (25 mM), and (only in case of **B** and **D**) OleD (125 µM). **E**) Color development during the synthesis of UDP-GlcNAz. 1: GlcNAz-CNP in buffer, 2: UDP in buffer, 3: 2-chloro-4-nitrophenol (CNP) in buffer, 4: The reaction mixture. Incubations were conducted at 37 ℃ overnight in 20 mL Tris-HCl buffer (50 mM, pH 8.0) with GlcNAz-CNP (5 mM), UDP (5 mM), MgSO4 (25 mM), and OleD (125 µM).

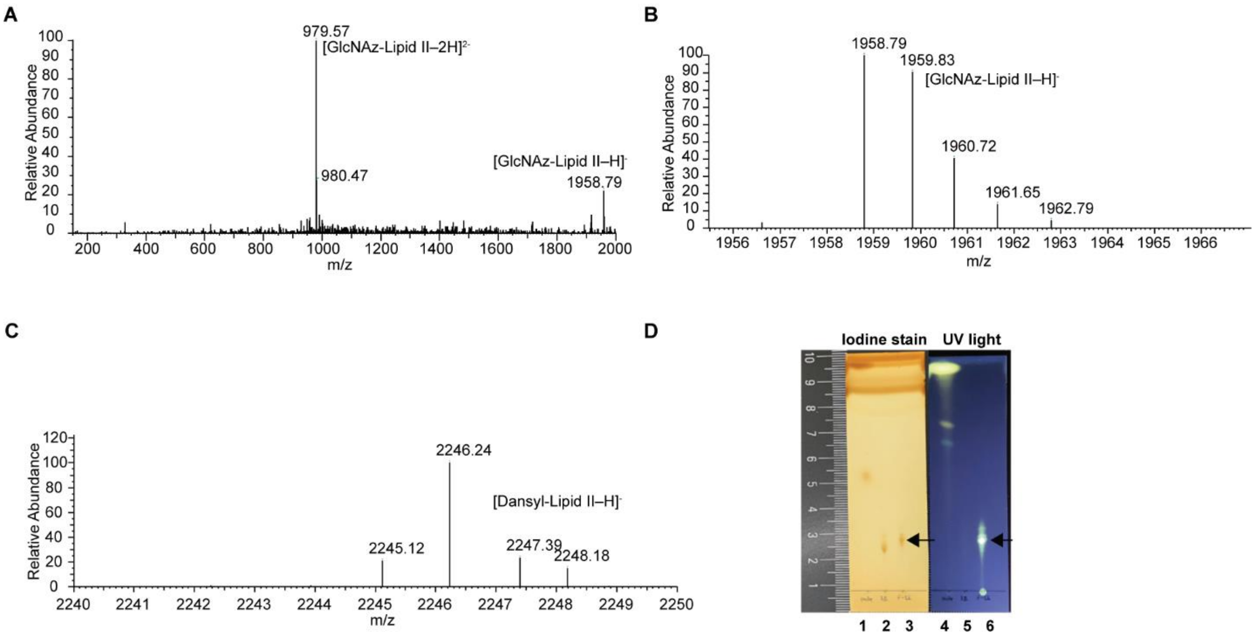

Figure S2. Mass spectrum in negative ion mode of GlcNAz-Lipid II and dansyl-GlcNAz-Lipid II and TLC analysis of the fluorescent Lipid II, related to Figure 3B. The *m*-Dap version of GlcNAz-Lipid II was synthesized by the isolation of membranes and dansyl-GlcNAz-Lipid II was generated by click chemistry (see methods). **A**) The mass of GlcNAz-Lipid II as determined in the negative mode is consistent with the expected value. **B**) The zoomed figure of the mass of GlcNAz-Lipid II with a loss of one proton. **C**) The zoomed figure of the mass of dansyl-GlcNAz-Lipid II with a loss of one proton. **D**) The TLC analysis of dansyl-GlcNAz-Lipid II. Lane 1 and lane 4: dansyl alkyne; lane 2 and lane 5: GlcNAz-labeled Lipid II; lane 3 and lane 6: The product of GlcNAz-labeled-Lipid II reacted with dansyl alkene (after 2 h). The left TLC plate is stained with iodine; the right TLC plate is under a UV_{365nm} lamp. The product was stained and observed as the black arrows show.

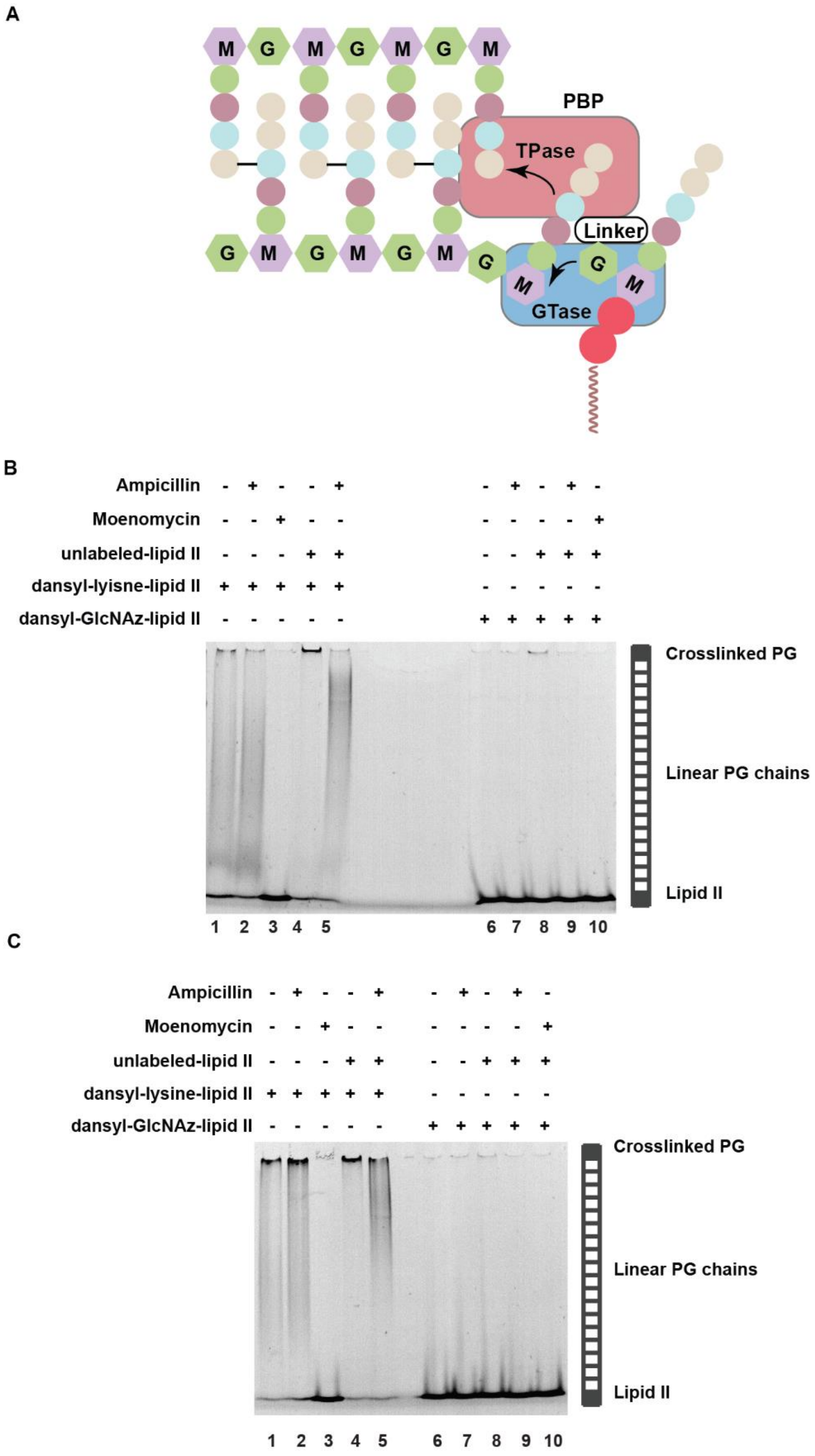

Figure S3. Scheme of peptidoglycan synthesis by bifunctional PBPs and SDS-PAGE analysis of PG products, related to Figure 4. A) Scheme of peptidoglycan synthesis by bifunctional PBPs. Bifunctional class A penicillin-binding proteins (PBPs)

can catalyze both GTase and TPase reactions. GTase can polymerize the disaccharide-pentapeptide into glycan chains, and TPase catalyzes cross-linking between stem peptides. **B**) SDS-PAGE analysis of PG products of the dansyl-lysine-Lipid II and dansyl-GlcNAz-Lipid II reactions by *P. aeruginosa* PBP1B. Samples were incubated for 2 h at 37°C and boiled. All reactions contain *P. aeruginosa* PBP1B (0.5 µM), LpoP (2 µM), and dansyl-lysine-Lipid II (lanes 1-5) or dansyl-GlcNAz-Lipid II (lanes 6-10) (each 10 µM). For the reaction with dansyl-GlcNAz-Lipid II, some crosslinked PG was produced in the presence of unlabeled-Lipid II (lane 8) which can be abolished by the addition of ampicillin (lane 10), whereas no crosslinked PG was produced in absence of unlabeled Lipid II (lanes 6 and 7). In contrast, for the reaction with dansyl-lysine-Lipid II, a much more crosslinked PG was produced in the presence of unlabeled Lipid II (lane 4). Crosslinked and linear PG chains were also produced in absence of unlabeled Lipid II (lane 1). The crosslinked and linear PG chains can't be abolished by the addition of ampicillin (lanes 2 and 5). The addition of moenomycin abolished the appearance of crosslinked and linear PG chains in the reaction with either dansyl-lysine-Lipid II or dansyl-GlcNAz-Lipid II (lanes 3 and 10). The results show that the incorporation of dansyl-GlcNAz-Lipid II by *P. aeruginosa* PBP1B is because of the TPase and not the GTase, whereas the incorporation of dansyl-lysine-Lipid II is due to the GTase. **C**) SDS-PAGE analysis of PG products of the dansyl-lysine-Lipid II and dansyl-GlcNAz-Lipid II reactions by *E. coli* PBP1A. Samples were incubated for 2 h at 37°C and boiled. All reactions contain *E. coli* PBP1A (0.7 µM) and LpoA (2.8 µM), and dansyl-lysine-Lipid II (lanes 1-5) or dansyl-GlcNAz-Lipid II (lanes 6-10) (each 10 µM). For the reaction with dansyl-GlcNAz-Lipid II, no crosslinked PG was produced either in the presence with unlabeled-Lipid II (lanes 8-10) or absence of unlabeled Lipid II (lanes 6 and 7). In contrast, for the reaction with dansyl-lysine-Lipid II, crosslinked and linear PG chains were also produced in the presence of Lipid II (lane 4) or the absence of unlabeled Lipid II (lane 1). The crosslinked and linear PG chains can't be abolished by the addition of ampicillin (lanes 2 and 5). The addition of moenomycin abolished the appearance of crosslinked and linear PG chains (lane 3). The results show that *E. coli* PBP1A does not incorporate dansyl-GlcNAz-Lipid II in these conditions with either the GTase or the TPase.

A

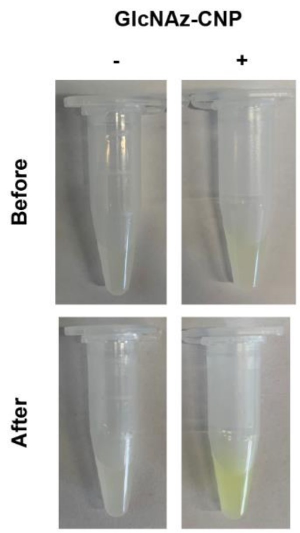

B

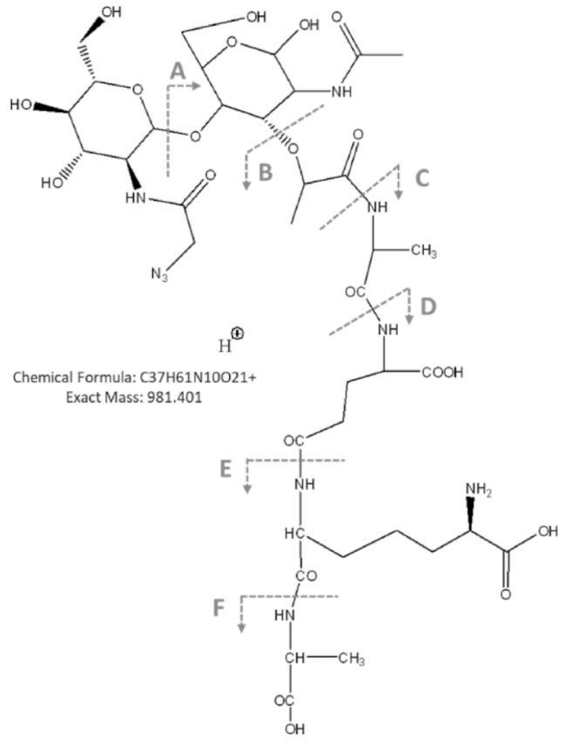

C

Ion Peak (rank intensity)	Observed m/z	Ion Fragment Containing
1	302.1521	[D to E]$^+$
2	534.2652	[B to F]$^+$
3	391.2012	[D to F]$^+$
4	262.1563	[E to F]$^+$
5	445.2124	[B to E]$^+$
6	963.4217	[Parent ion – H_2O]$^+$
7	719.3369	[A to F – H_2O]$^+$
8	981.4425	[Parent ion]$^+$
9	737.3457	[A to F]$^+$

Figure S4. Colorimetric analysis of *in vivo* labeling and structure of GlcNAz-modified disaccharide tetrapeptide monomer and the detected masses of its fragment ions, related to Figure 5. A) The colorimetric analysis of the LB medium of BL21 (pET28a-OleD-TDP16) before and after incubation with GlcNAz-CNP. BL21 (pET28a-OleD-TDP16) grew in LB medium (containing 50 µg mL^{-1} kanamycin) with (+, right) or without (-, left) GlcNAz-CNP (0.2%) and 0.4 mM IPTG for 2 h. After bacteria grew for 2 h, the color of the growth medium containing GlcNAz-CNP became yellowish, while the color of the group in the medium containing no GlcNAz-CNP did not change. **B**) Structure of the non-reduced, GlcNAz-modified disaccharide tetrapeptide monomer

released from PG by cellosyl. Predicted fragmentations are indicated with dotted lines. Arrowheads indicate the residual fragment ion composition. Substructures are labeled A to F for the disaccharide tetrapeptide, and the detected masses of fragment ions (Figure 5 D) are listed in **C**).

Supplemental Schemes:

Scheme 1-4 Synthesis of compounds **1-4**. Related to STAR★Methods Synthesis of compounds

1.

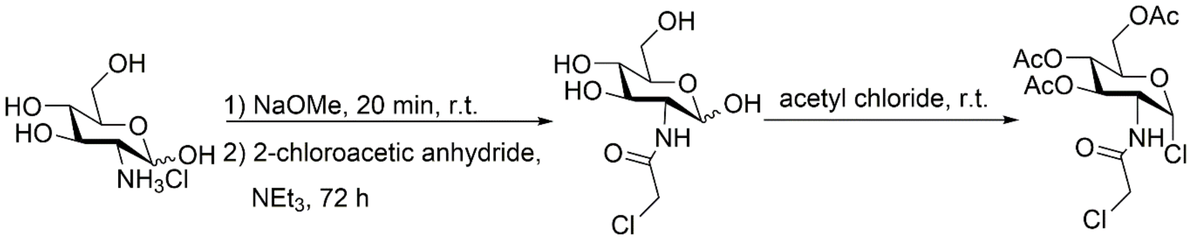

1

2.

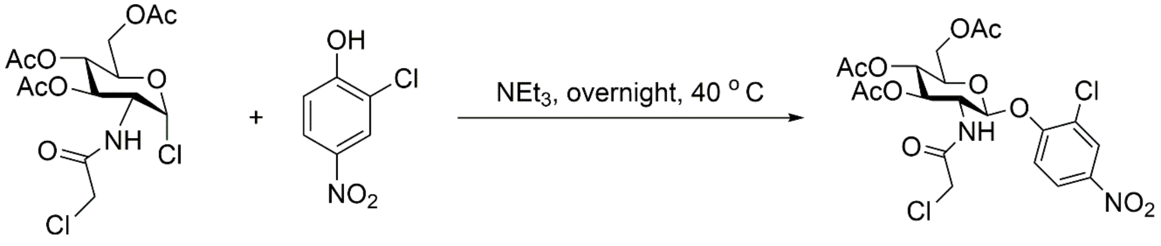

1 **2**

3.

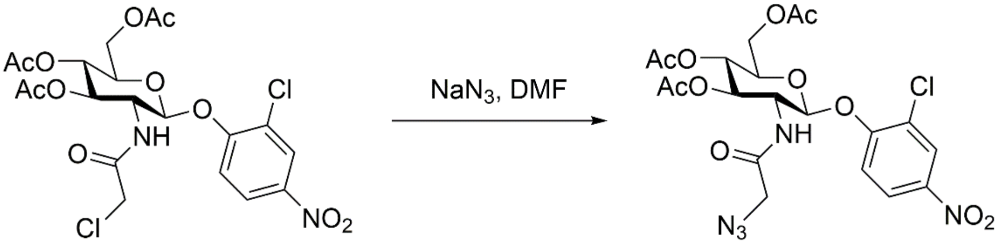

2 **3**

4.

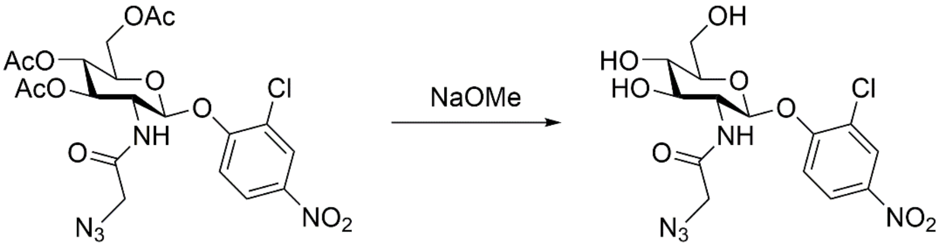

3 **4**
(GlcNAz-CNP)

Data S1. NMR spectra of compound 1, related to the STAR Methods section.

^{1}H NMR of compound **1** (400 MHz, in $CDCl_3$)

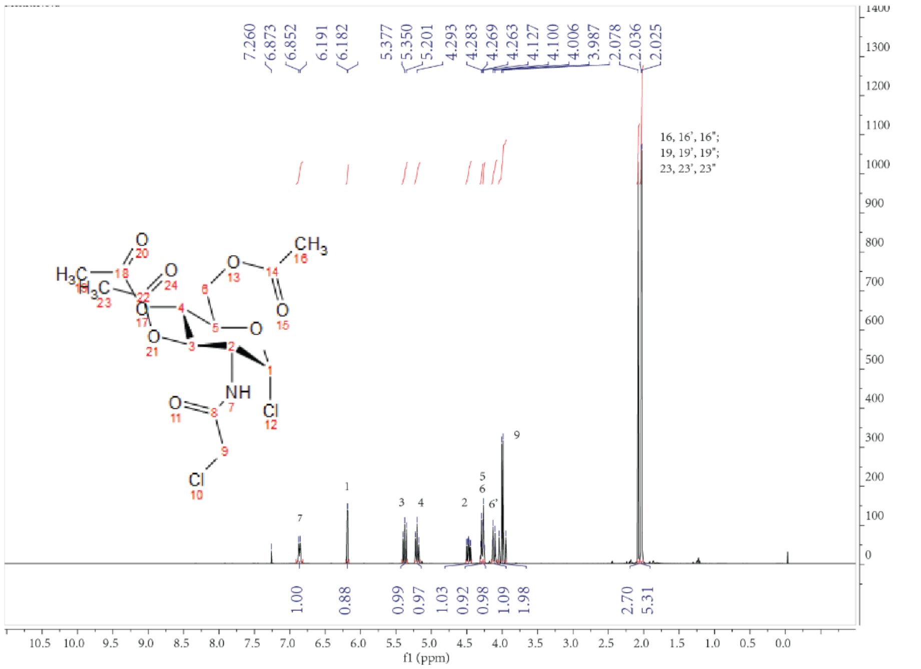

^{1}H NMR of compound **1** (400 MHz, in $CDCl_3$) (zoomed)

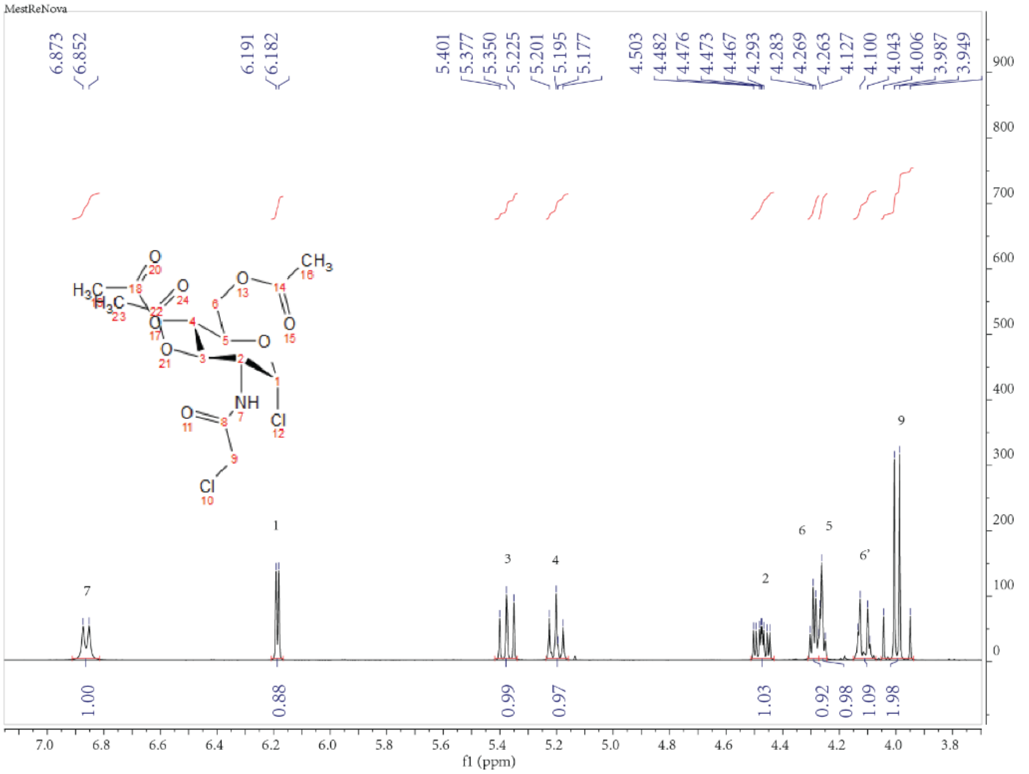

^{13}C NMR of compound **1** (101 MHz, in $CDCl_3$)

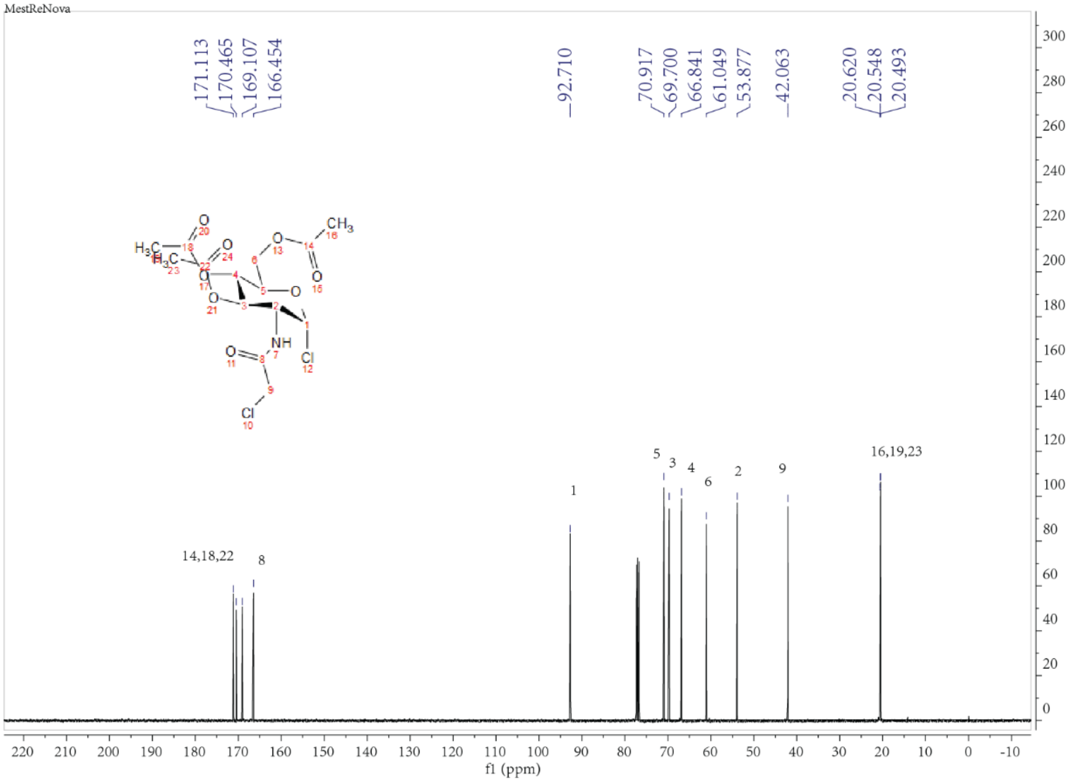

H-H COSY of compound **1** (in $CDCl_3$)

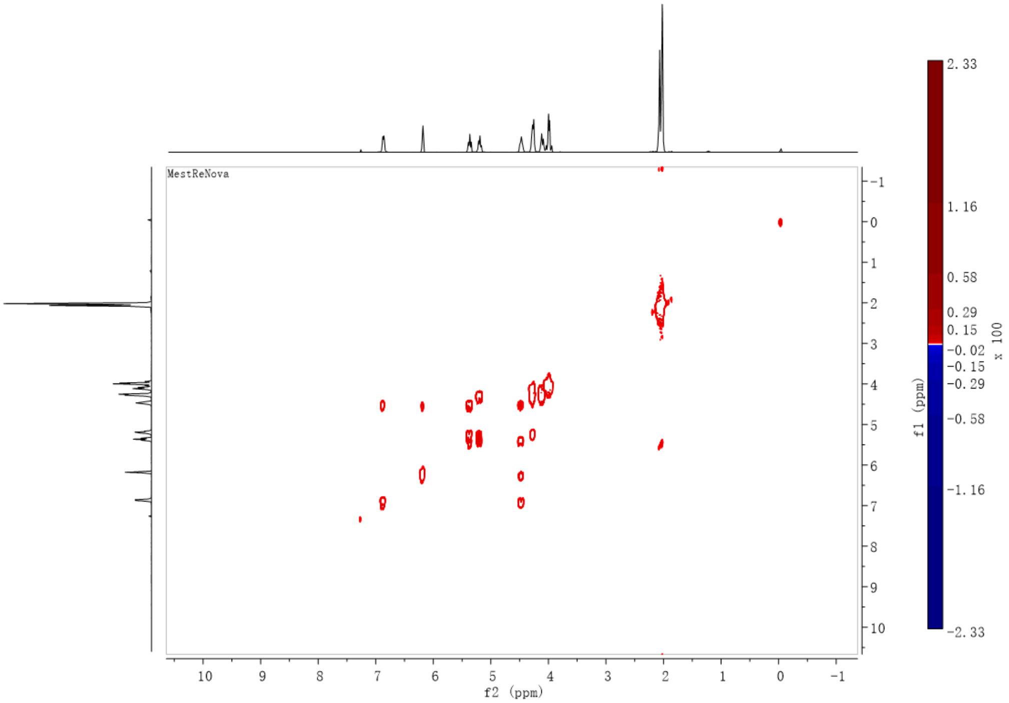

C-H HSQC of compound **1** (in $CDCl_3$) (blue spots for negative phase and red spots for positive phase)

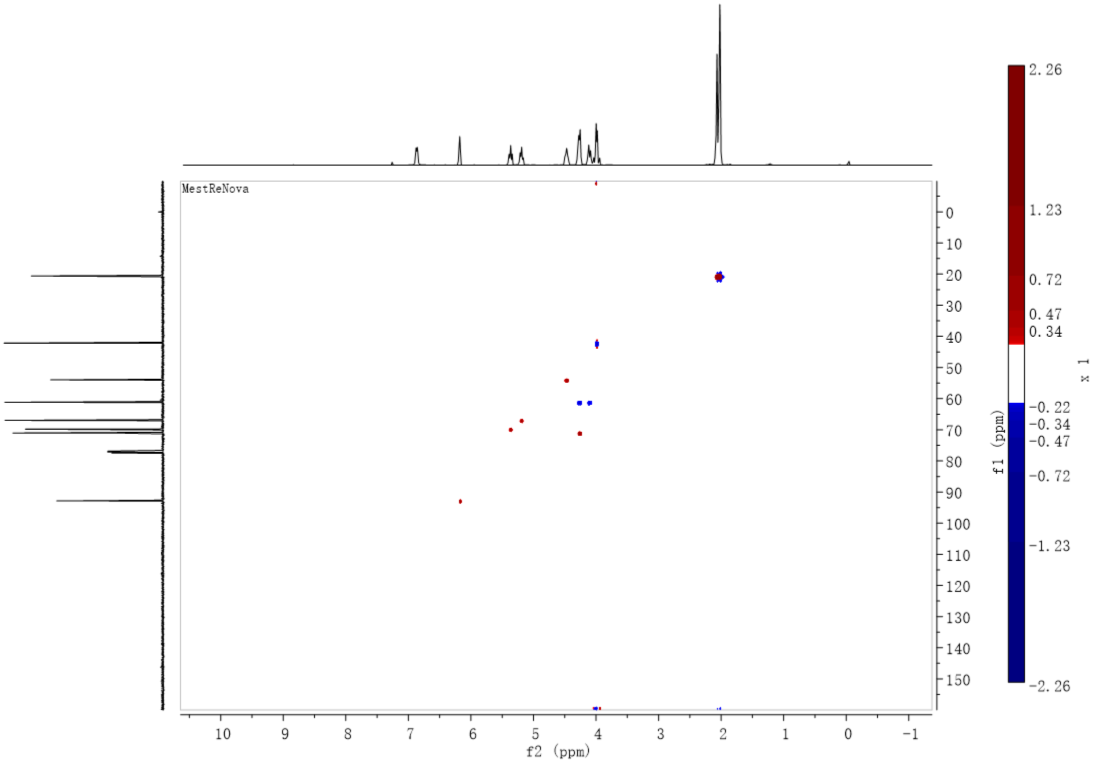

Data S2. NMR spectra of compound 2, Related to the STAR Methods section.

^{1}**H NMR** of compound **2** (400 MHz, in $CDCl_3$)

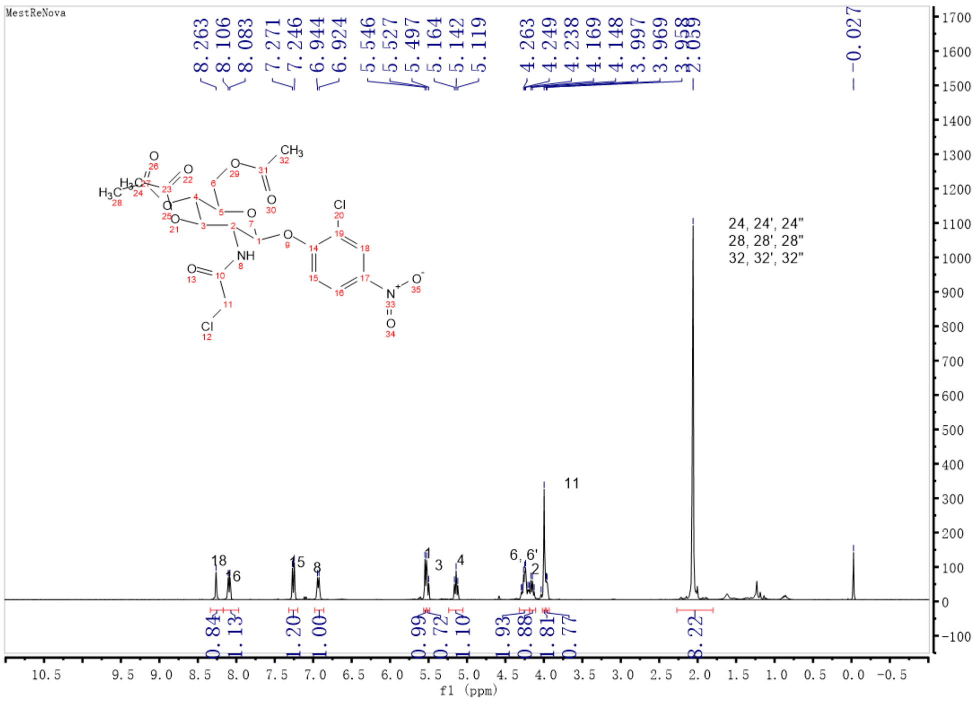

^{1}**H NMR** of compound **2** (400 MHz, in $CDCl_3$) (zoomed)

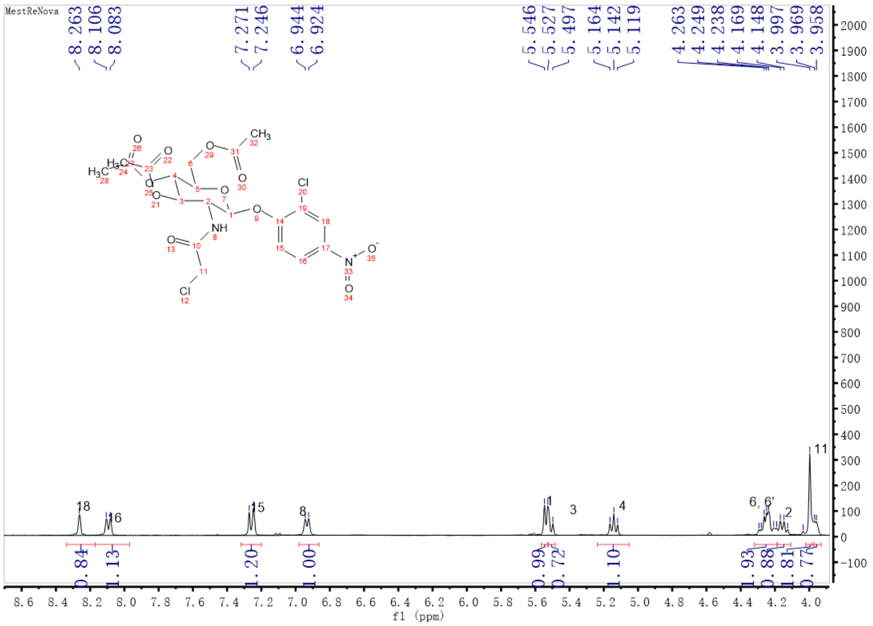

^{13}**C NMR** of compound **2** (101 MHz, in $CDCl_3$)

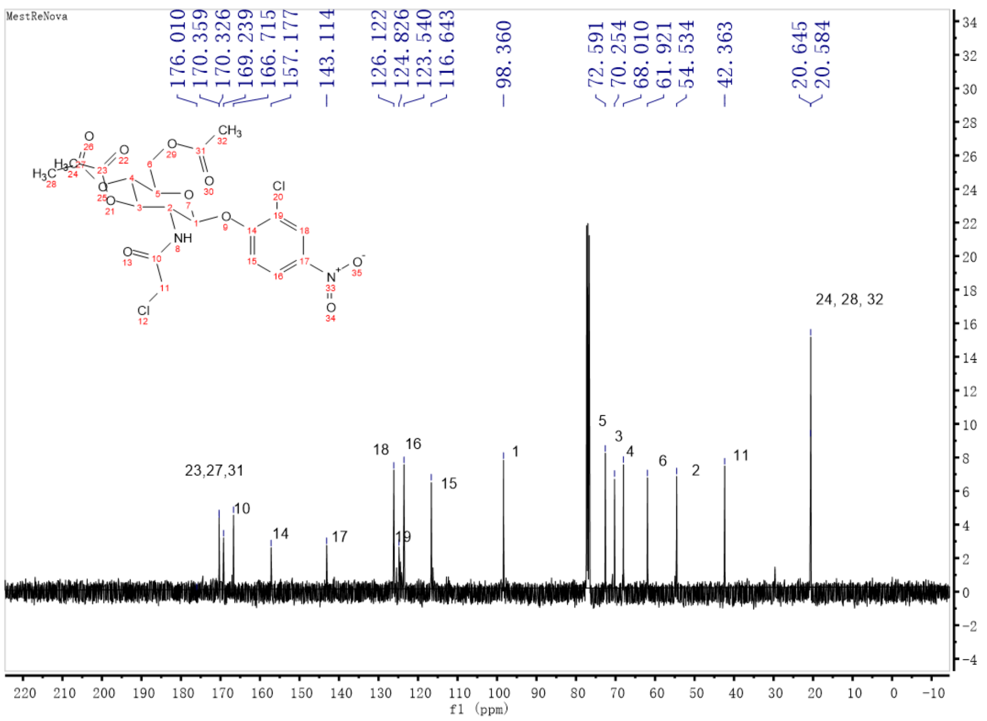

H-H **COSY** of compound **2** (in CDCl3)

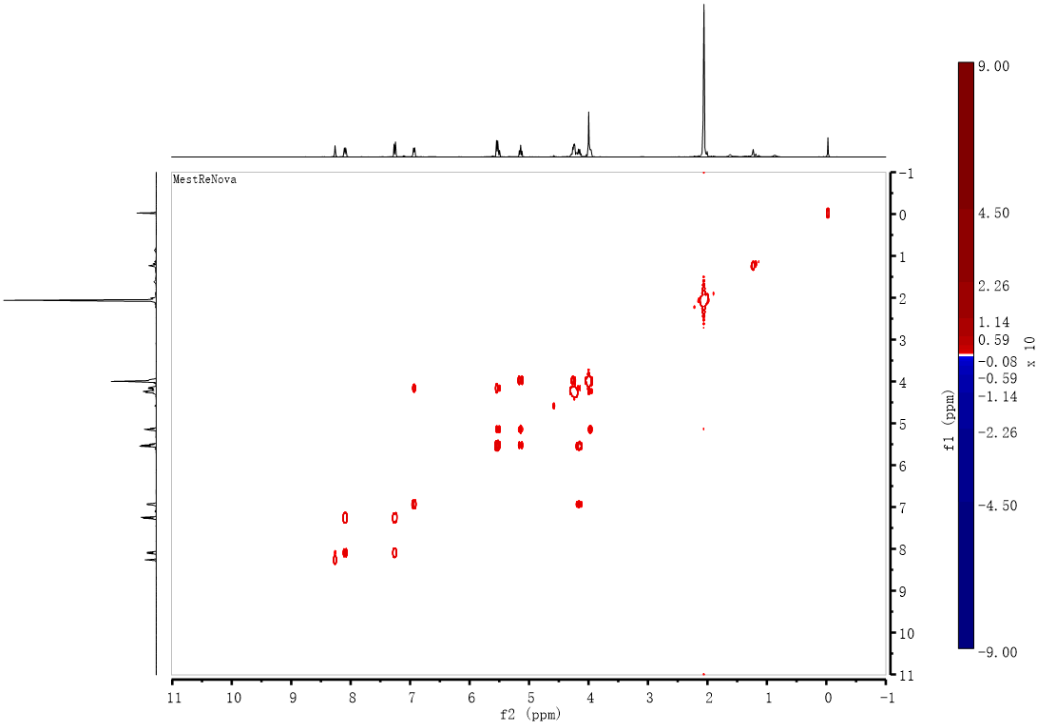

C-H **HSQC** of compound **2** (in CDCl3) (blue spots for negative phase and red spots for positive phase)

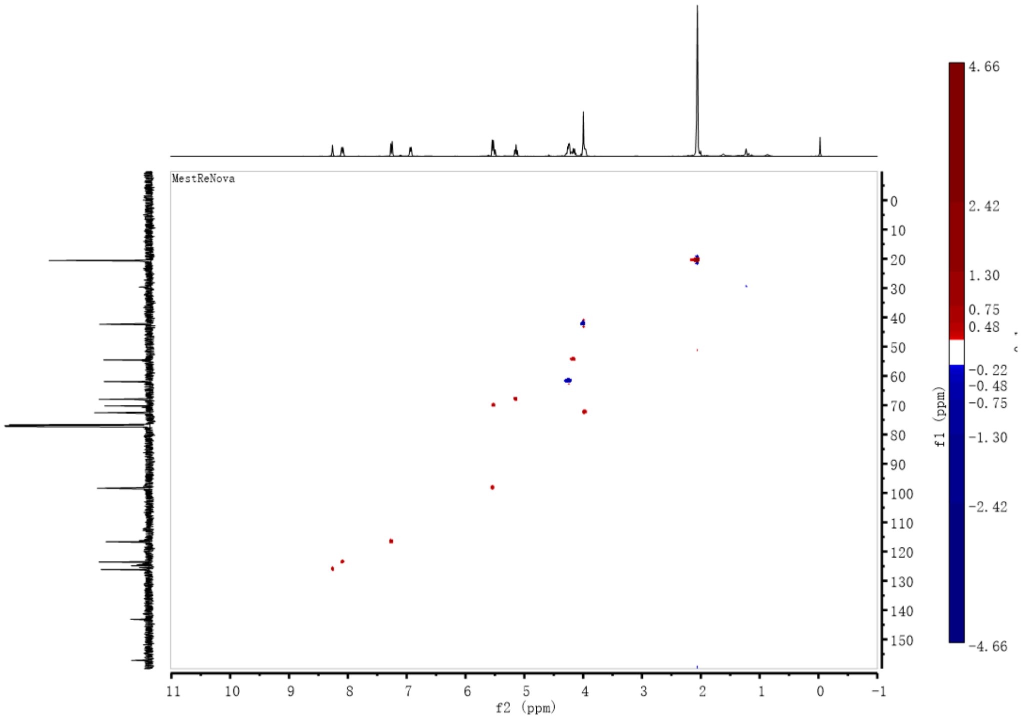

MestReNova
f2 (ppm)
f1 (ppm)

Data S3. NMR spectra of compound 3, Related to the STAR Methods section.

^{1}H NMR of compound **3** (400 MHz, in $CDCl_3$)

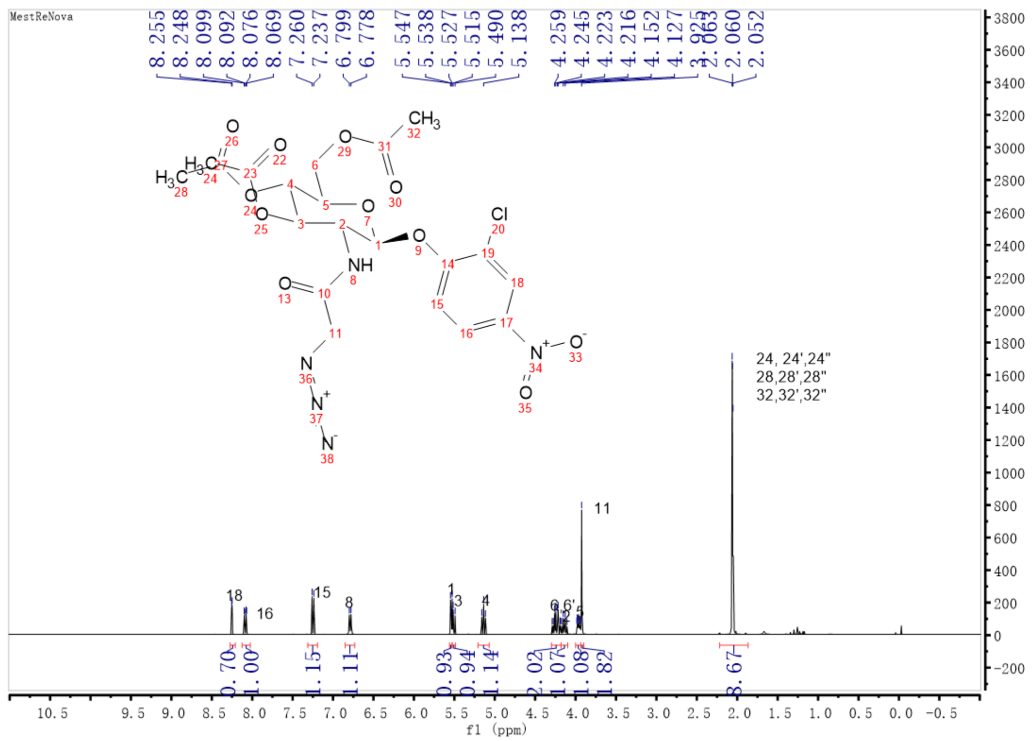

^{1}H NMR of compound **3** (400 MHz, in $CDCl_3$) (zoomed)

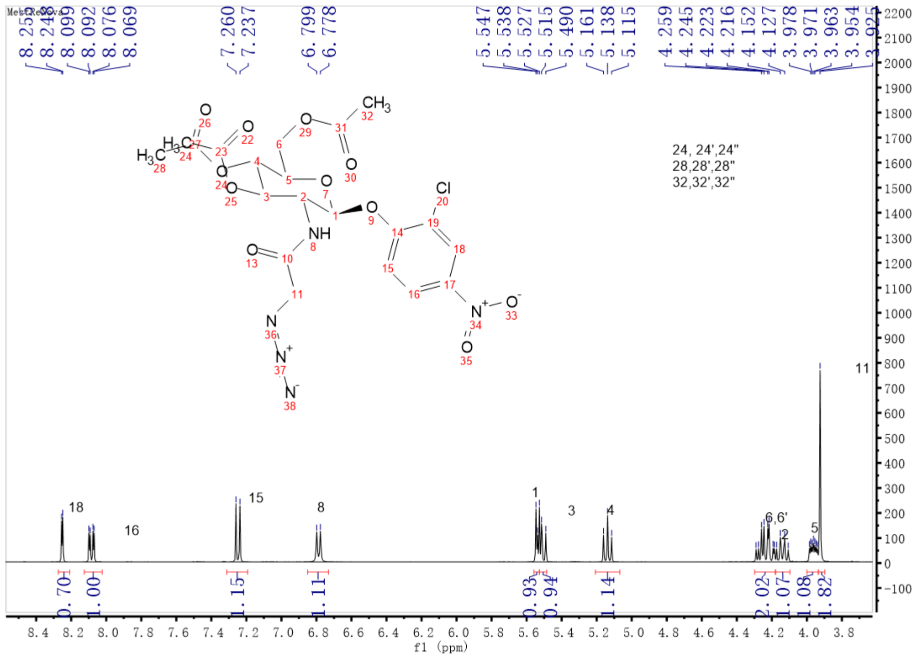

^{13}C NMR of compound **3** (101 MHz, in $CDCl_3$)

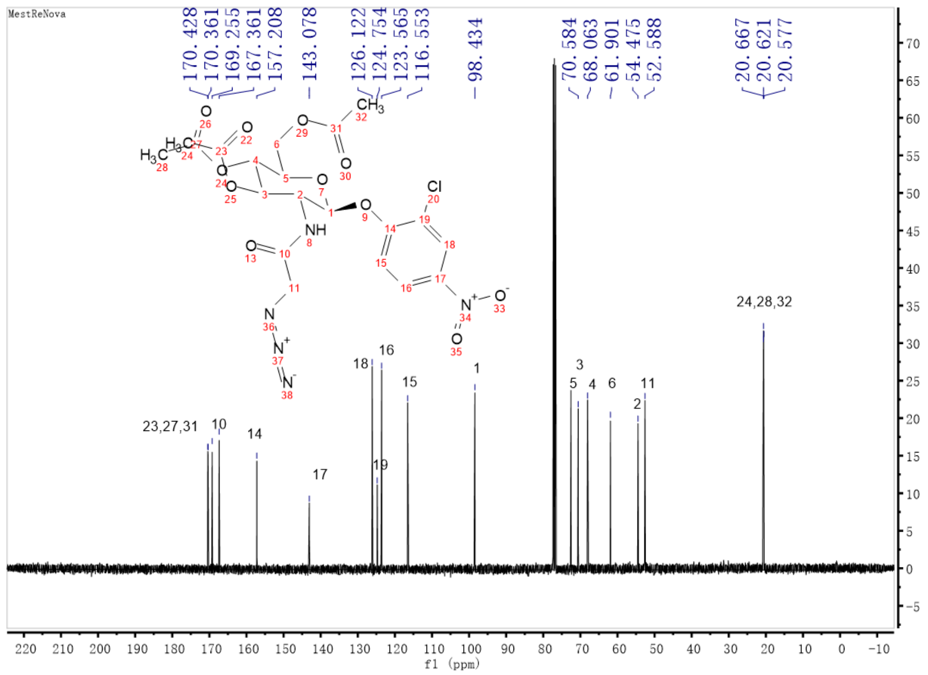

H-H COSY of compound **3** (in $CDCl_3$)

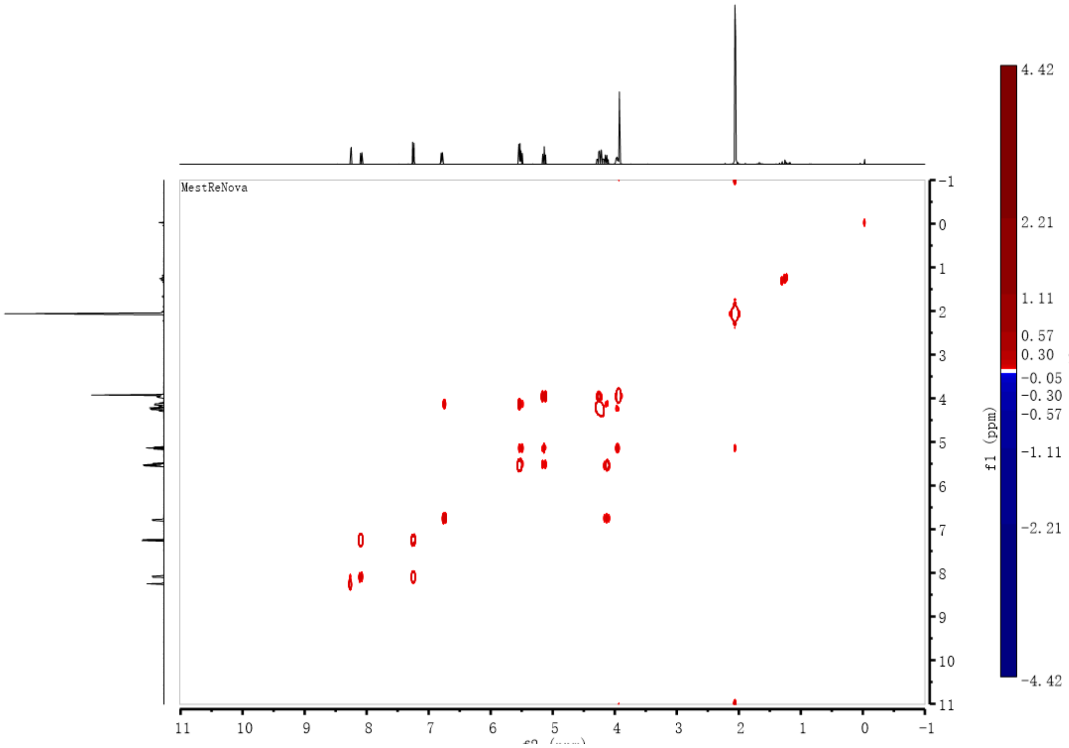

C-H HSQC of compound **3** (in $CDCl_3$) (blue spots for negative phase and red spots for positive phase)

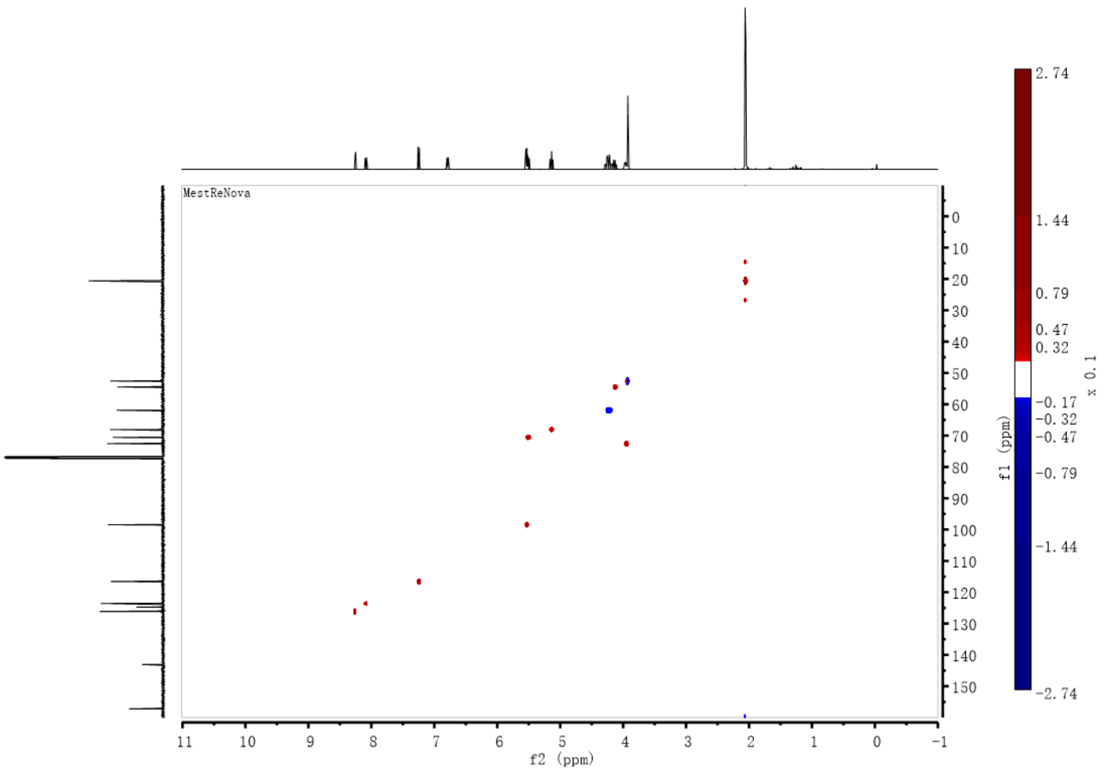

Data S4. NMR spectra of compound 4, Related to the STAR Methods section.

^{1}H NMR of compound **4** (**GlcNAz-CNP**) (400 MHz, in dimethyl sulfoxide-d_6)

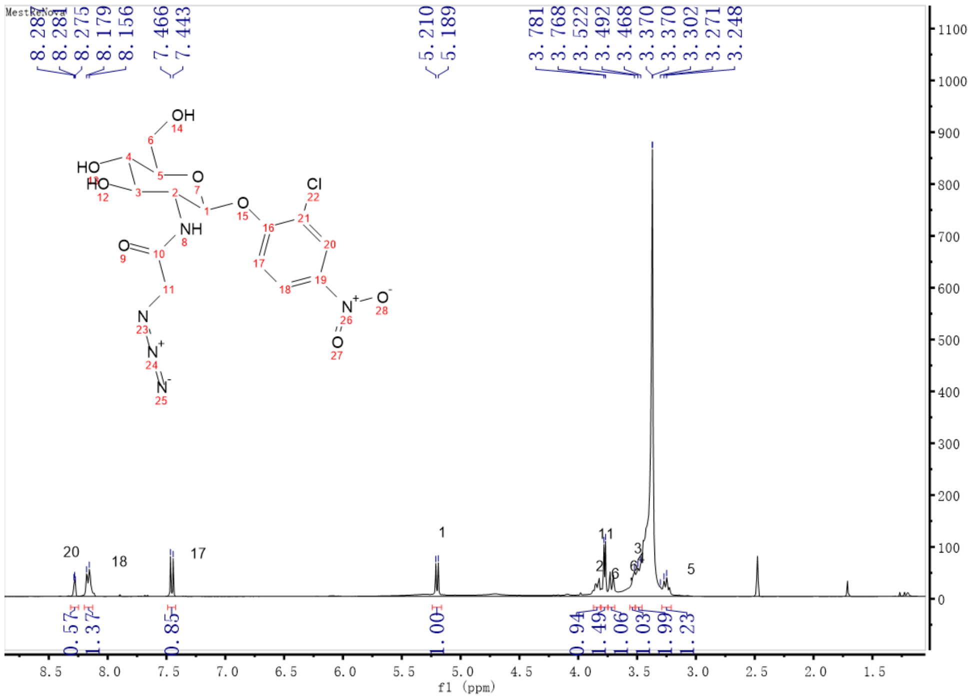

^{1}H NMR of compound **4** (**GlcNAz-CNP**) (400 MHz, in dimethyl sulfoxide-d_6) (zoomed)

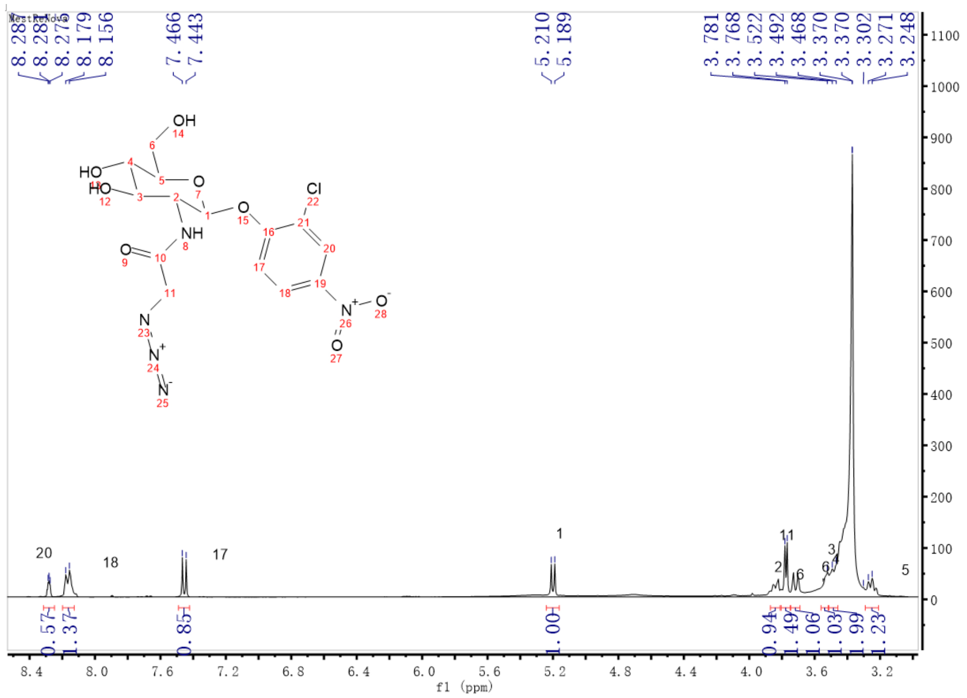

^{13}C NMR of compound **4** (**GlcNAz-CNP**) (101 MHz, in dimethyl sulfoxide-d$_6$)

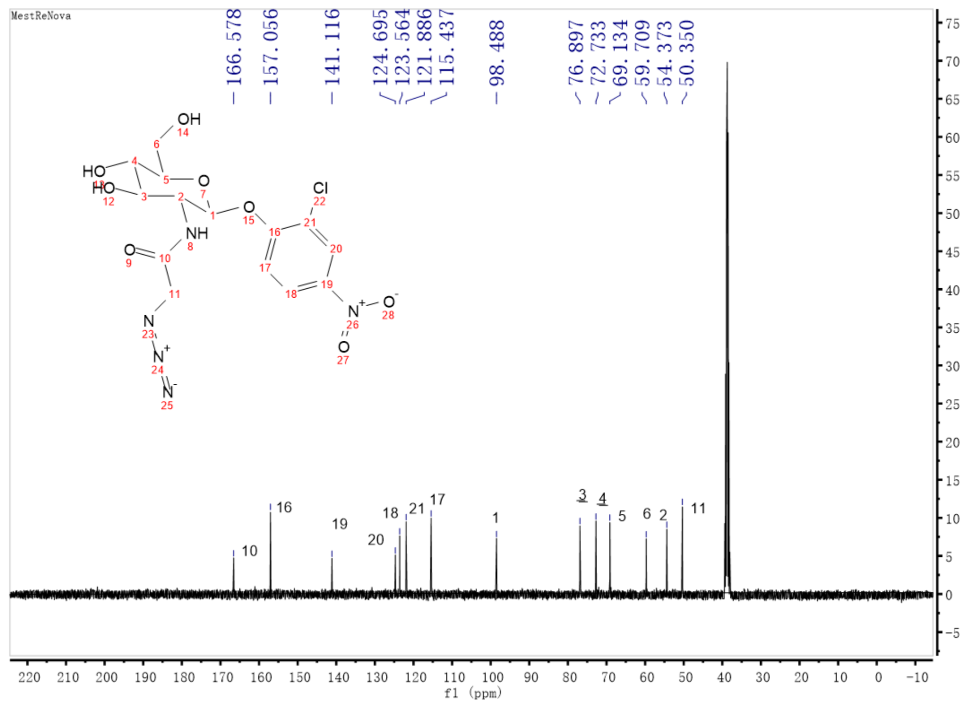

H-H COSY of compound **4** (**GlcNAz-CNP**) (in dimethyl sulfoxide-d$_6$)

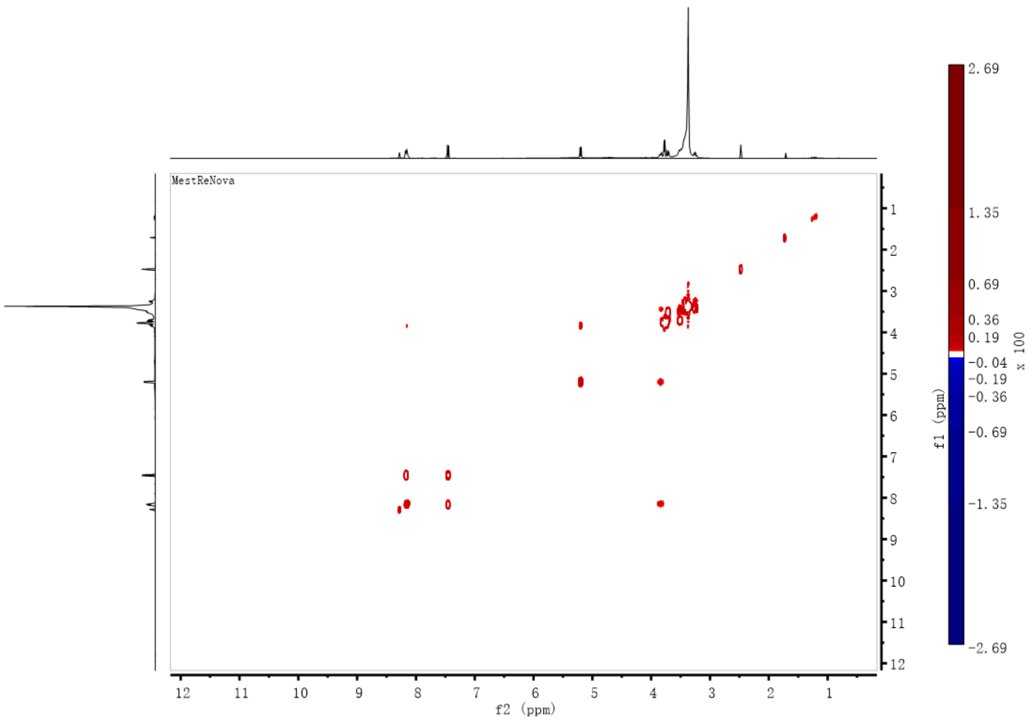

C-H HSQC of compound **4** (**GlcNAz-CNP**) (in dimethyl sulfoxide-d_6) (blue spots for negative phase and red spots for positive phase)

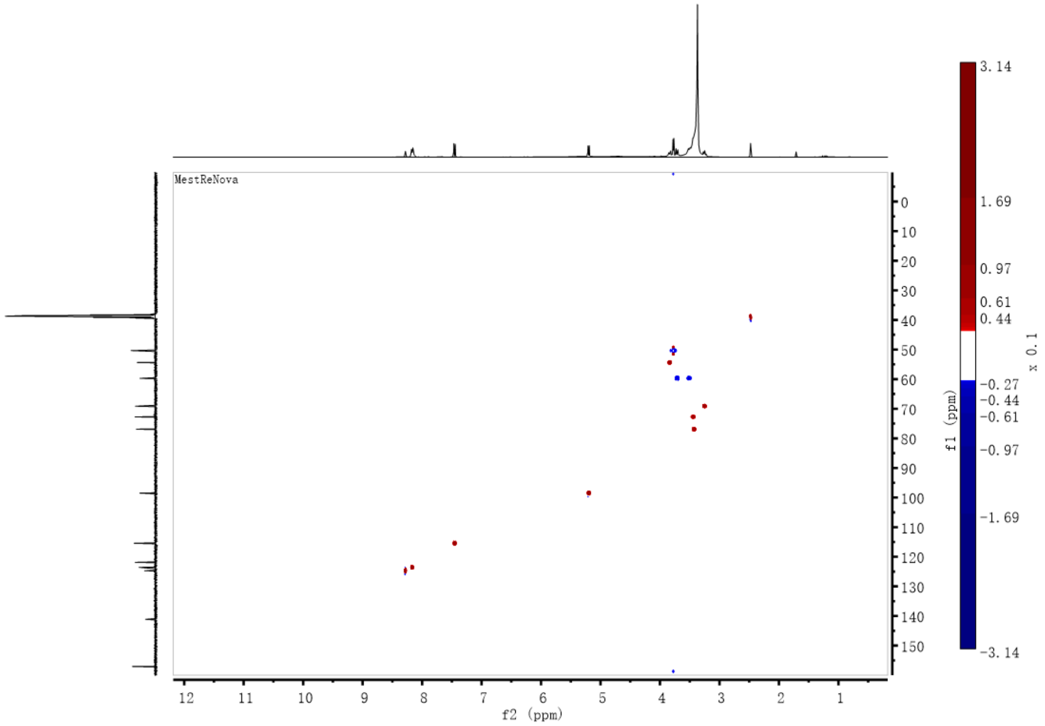

Data S5. NMR spectra of compound UDP-GlcNAz, Related to the STAR Methods section.

^{1}H NMR of **UDP-GlcNAz** (400 MHz, in D_2O)

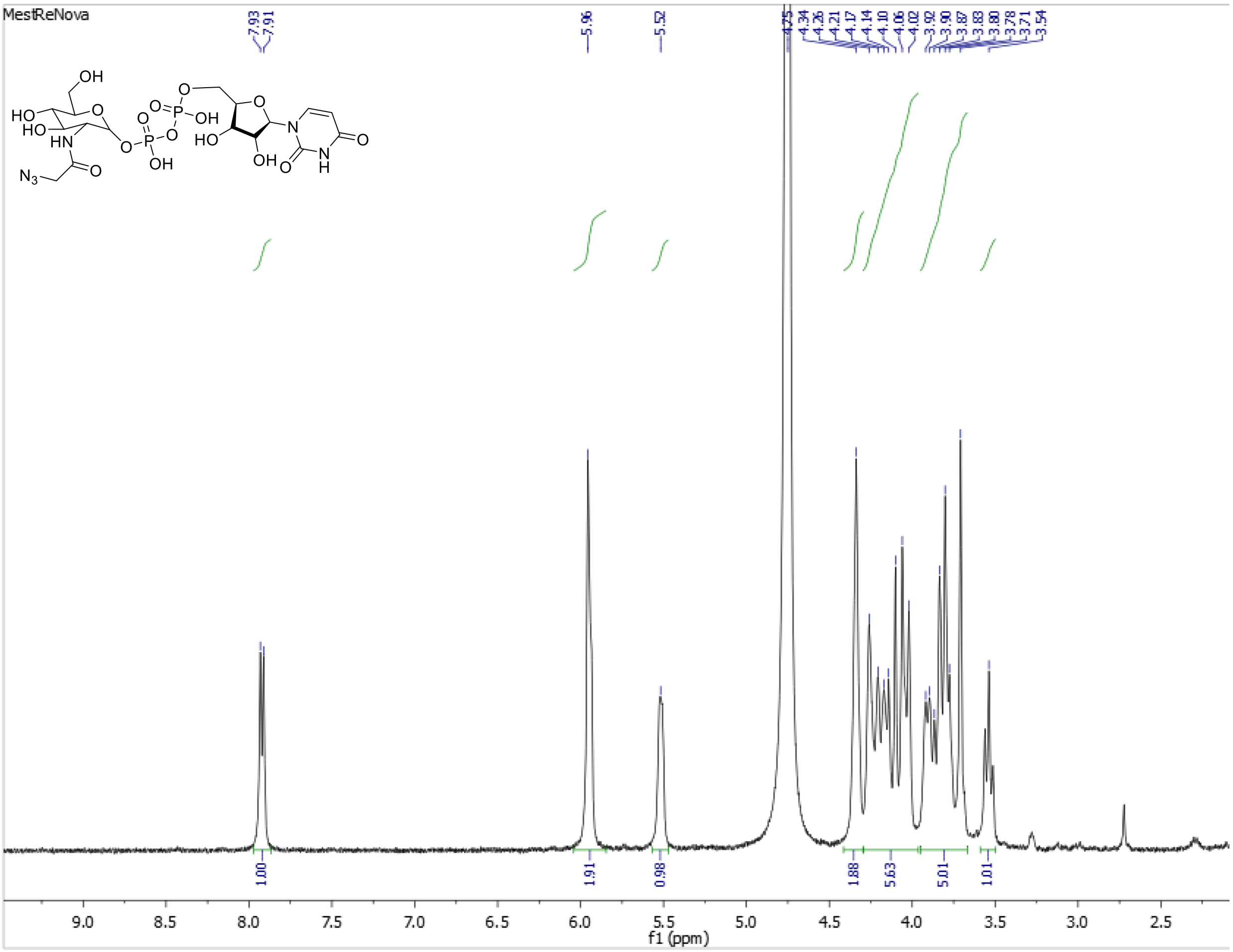

^{13}C NMR of **UDP-GlcNAz** (101 MHz, in D_2O)

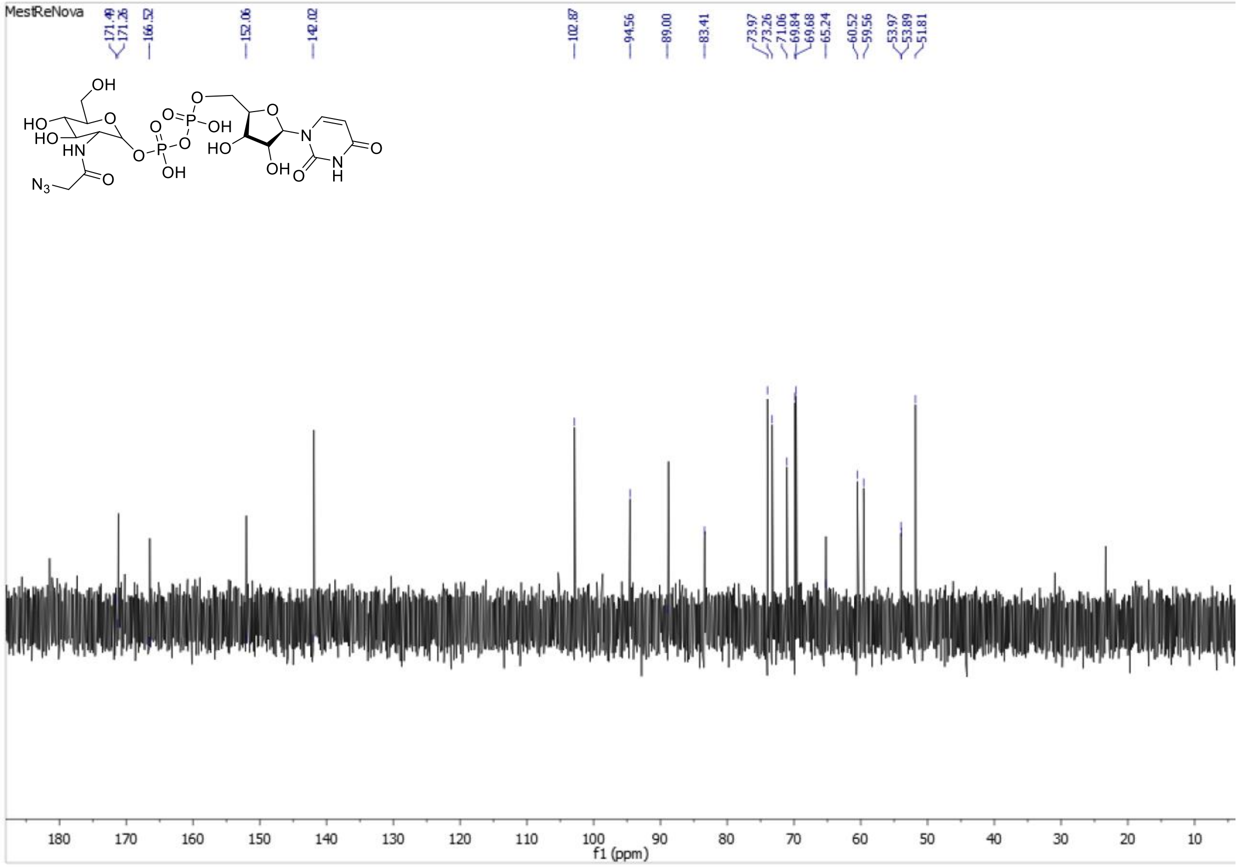

^{31}P NMR of **UDP-GlcNAz** (in D_2O)

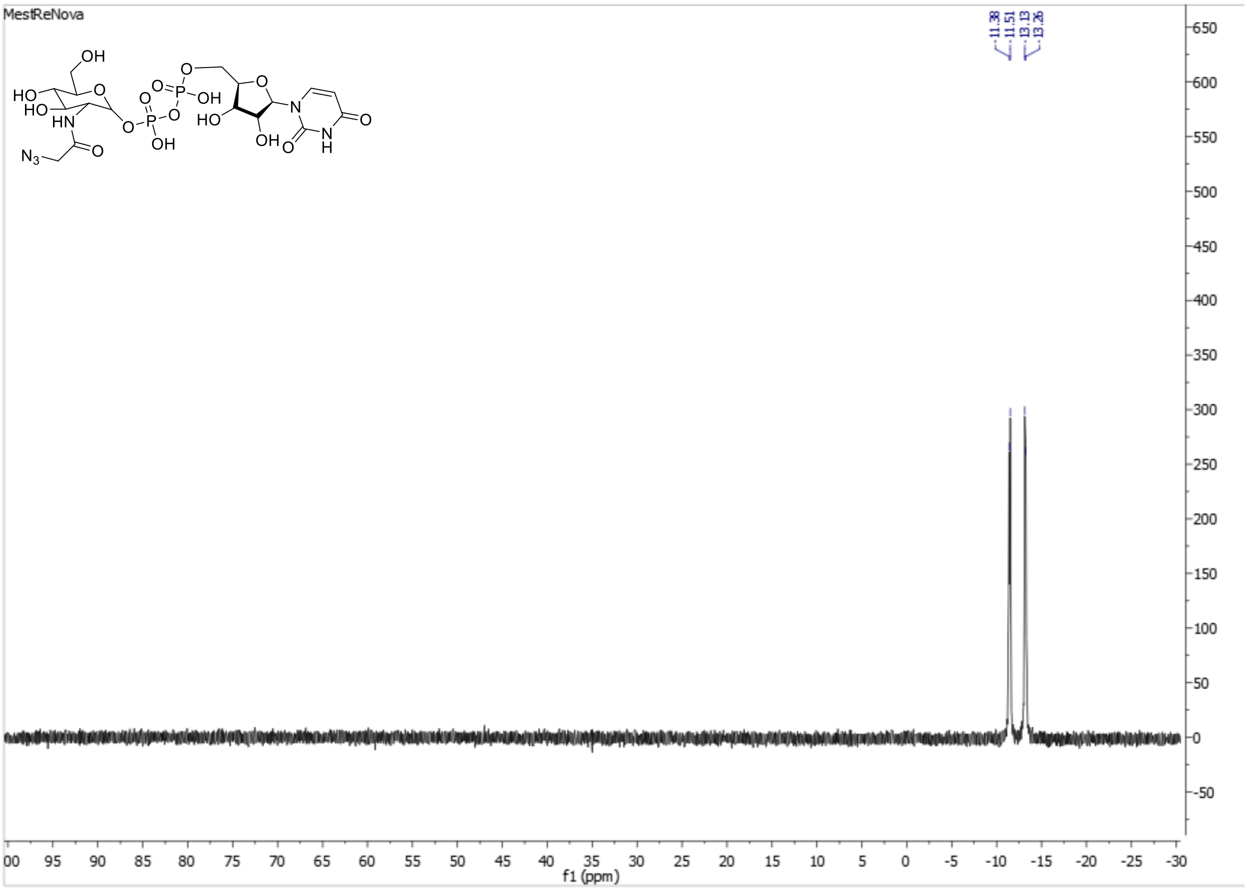

H-H COSY of **UDP-GlcNAz** (in D_2O)

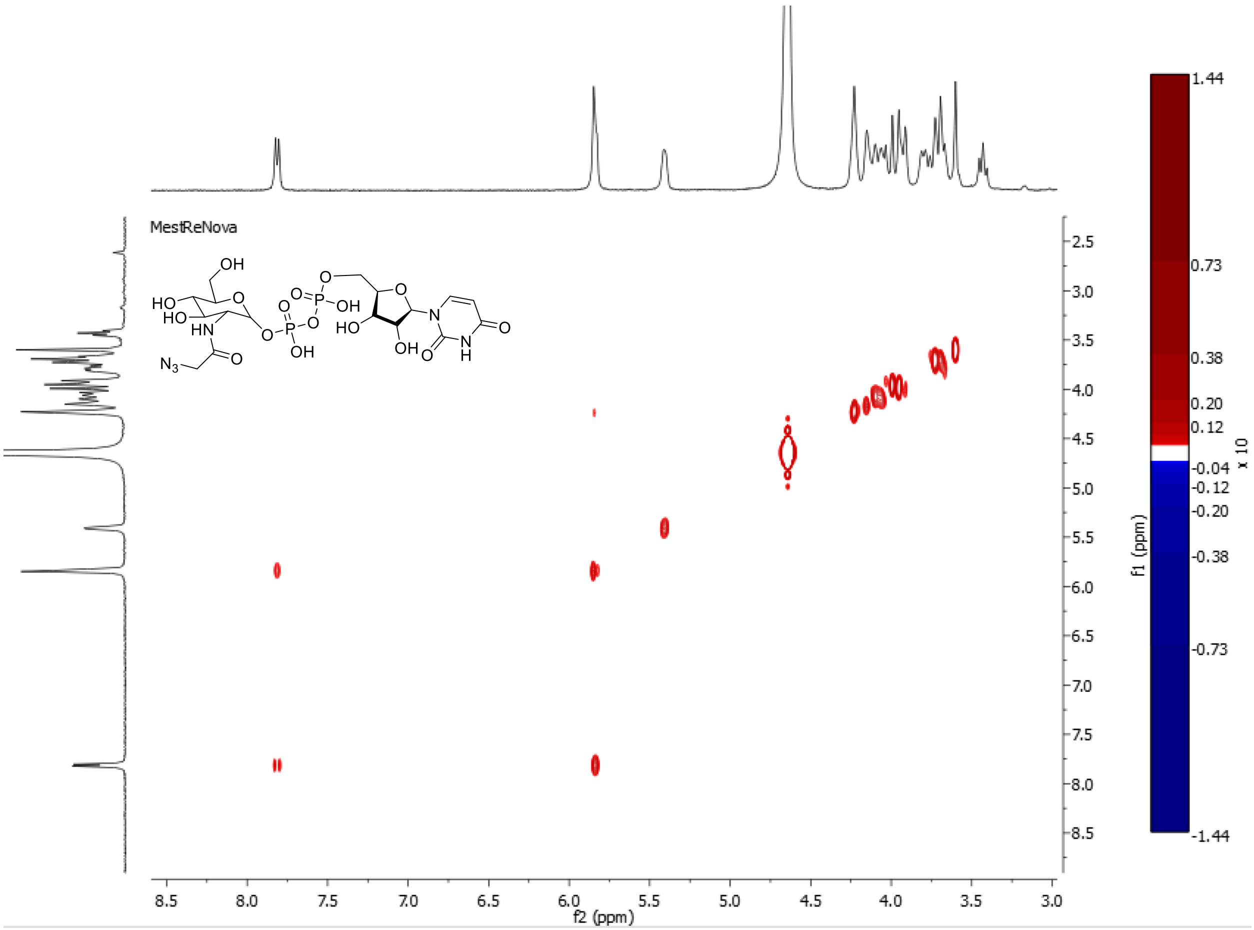

H-C HSQC of **UDP-GlcNAz** (in D_2O)

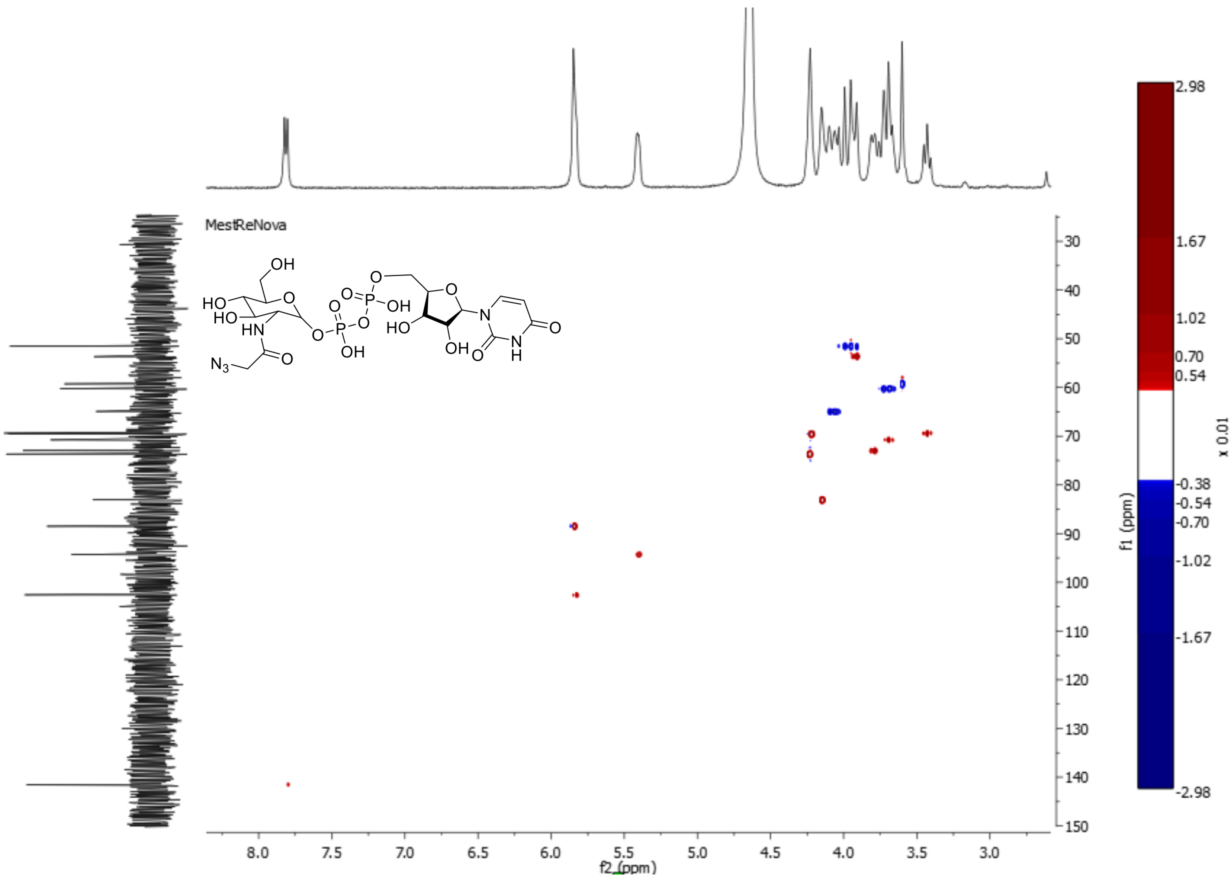

Supplement: Document S1. Figures S1–S4, Schemes S1–S4 and Data S1–S5 [file mmc1.pdf]
